# Reductive Supramolecular In Situ Construction of Nano‐Platinum Effectively Couples Cathodic Hydrogen Evolution and Anodic Alcohol Oxidation

**DOI:** 10.1002/advs.202502002

**Published:** 2025-04-03

**Authors:** Rui Bai, Qiao Ye, Cuiyu Li, Haijian Wang, Yan Zhao, Yicheng Zhang, Yingtang Zhou, Xue Zhao

**Affiliations:** ^1^ Yunnan Key Laboratory of Modern Separation Analysis and Substance Transformation College of Chemistry and Chemical Engineering Yunnan Normal University Kunming 650500 P.R. China; ^2^ Zhejiang Key Laboratory of Petrochemical Environmental Pollution Contro National Engineering Research Center for Marine Aquaculture Marine Science and Technology College Zhejiang Ocean University Zhoushan 316004 P.R. China

**Keywords:** hydrogen evolution, in situ reduction, methanol oxidation, nano‐Pt, reductive supermolecular

## Abstract

The deployment of high‐performance catalysts and the acceleration of anodic reaction kinetics are key measures to achieve maximum energy efficiency in overall water electrolysis hydrogen production systems. Here, an innovative strategy is developed by directly constructing a supramolecular framework embedded with boron clusters and cucurbituril as reducing agent. This approach enabled the in situ conversion of Pt⁴⁺ into highly dispersed, small‐sized nano‐platinum, which are subsequently distributed on a boron‐carbon‐nitrogen (BCN) matrix. The resulting Pt/BNHCSs catalyst demonstrates the ability to facilitate electrocatalytic water splitting for hydrogen production across multiple scenarios while simultaneously accelerating the anodic methanol oxidation kinetics, significantly outperforming commercial Pt/C catalyst in various aspects. The cathodic hydrogen evolution‐anodic methanol oxidation coupling system constructed using the Pt/BNHCSs greatly reduces the overall energy consumption of the electrolysis system. In situ attenuated total reflection Fourier transform infrared and online differential electrochemical mass spectrometry reveals that the catalyst interface enhances H₂O adsorption and promotes the CH₃OH→CO conversion process, and density functional theory calculations indicated that the BCN support facilitated the evolution of H₂O to H₂ and CH₃OH to CO, elucidating the mechanism by which Pt/BNHCSs simultaneously promoted hydrogen production and methanol oxidation.

## Graphical abstract

The supramolecular framework reductant is developed to realize the in situ construction of highly dispersed ultrafine nano‐Pt. The nano‐Pt supported on the BCN carrier realizes multi‐scenario electrocatalytic water hydrogen production, and establish a high‐performance cathode hydrogen evolution‐anode methanol oxidation coupling system, presenting a low‐energy vision for future water electrolysis hydrogen production.

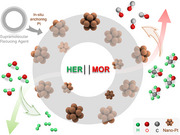

## 1 Introduction

Hydrogen is a green fuel with high energy density, and it hold great promise as a replacement for traditional fossil fuels once the challenges of production cost and storage are resolved.^[^ ^1^ ^]^ The development of water electrolysis for hydrogen production offers a promising pathway for green hydrogen generation, but it heavily relies on the use of high‐performance catalysts.^[^ ^2^ ^]^ Platinum‐based materials exhibit unparalleled activity in catalyzing the hydrogen evolution reaction (HER) and are currently the most widely used commercial catalysts.^[^ ^3^ ^]^ However, the high cost of platinum limits their potential for large‐scale applications, making the balance between cost and catalytic activity a critical challenge.^[^ ^3^, ^4^ ^]^

To continue leveraging the superior catalytic activity of platinum‐based materials for HER, improving the mass activity of platinum‐based catalysts and reducing cell voltage are feasible approaches at present.^[^ ^5^ ^]^ Over the past few years, extensive research has focused on the development of platinum‐based catalysts for HER, aiming to maximize atomic utilization of platinum by minimizing the size of platinum active sites and enhancing their dispersion. Wet chemical reduction methods (e.g., using sodium borohydride as a reducing agent), hydrothermal treatment, and impregnation‐pyrolysis techniques have been widely favored by researchers,^[^ ^6^ ^]^ leading to the development of various high‐performance HER catalysts, including single‐atom platinum, platinum alloys, and supported platinum nanoparticles. However, the existing preparation techniques for platinum‐based nanocatalysts still fall short of meeting the stringent requirements for catalytic activity, stability, and universality in HER.^[^ ^7^ ^]^ Therefore, advancing controllable preparation techniques for platinum‐based nanomaterials remains a crucial step in promoting the development of water electrolysis for hydrogen production.

Compared to cathodic HER, the oxygen evolution reaction (OER) occurring at the anode involves a four‐electron transfer process, and the high overpotential significantly increases the energy consumption of the overall electrolysis system.^[^ ^1^, ^8^ ^]^ This represents another major challenge for hydrogen production via water electrolysis. A reliable approach currently being explored is the rational utilization of anodic oxidation processes to simultaneously achieve value‐added products at the anode during cathodic hydrogen production, such as the electrooxidation of small organic molecules.^[^ ^9^ ^]^ The efficient coupling of cathodic hydrogen evolution with anodic electrooxidation requires the deployment of highly active bifunctional electrocatalysts, which has become a research hotspot in this field.

Here, we developed a unique and innovative strategy by preloading a modifiable boron cluster (*closo*‐[B_10_H_10_]^2−^) reducing agent into a supramolecular framework composed of cucurbituril (CB[7]), thereby constructing an in situ reduction platform to achieve the precise formation of highly dispersed, ultrasmall platinum nanoparticles. Ultimately, this study successfully obtained a bifunctional catalyst (Pt/BNHCSs) capable of efficiently catalyzing both the hydrogen evolution reaction and methanol oxidation reaction. A high‐performance cathodic hydrogen evolution‐anodic alcohol oxidation coupling system was established, demonstrating superior performance compared to commercial platinum‐carbon catalysts across multiple application scenarios. These results highlight the advancement of using reducing agent‐preloaded supramolecular frameworks for the precise construction of platinum‐based functional materials.

**Figure 1:**
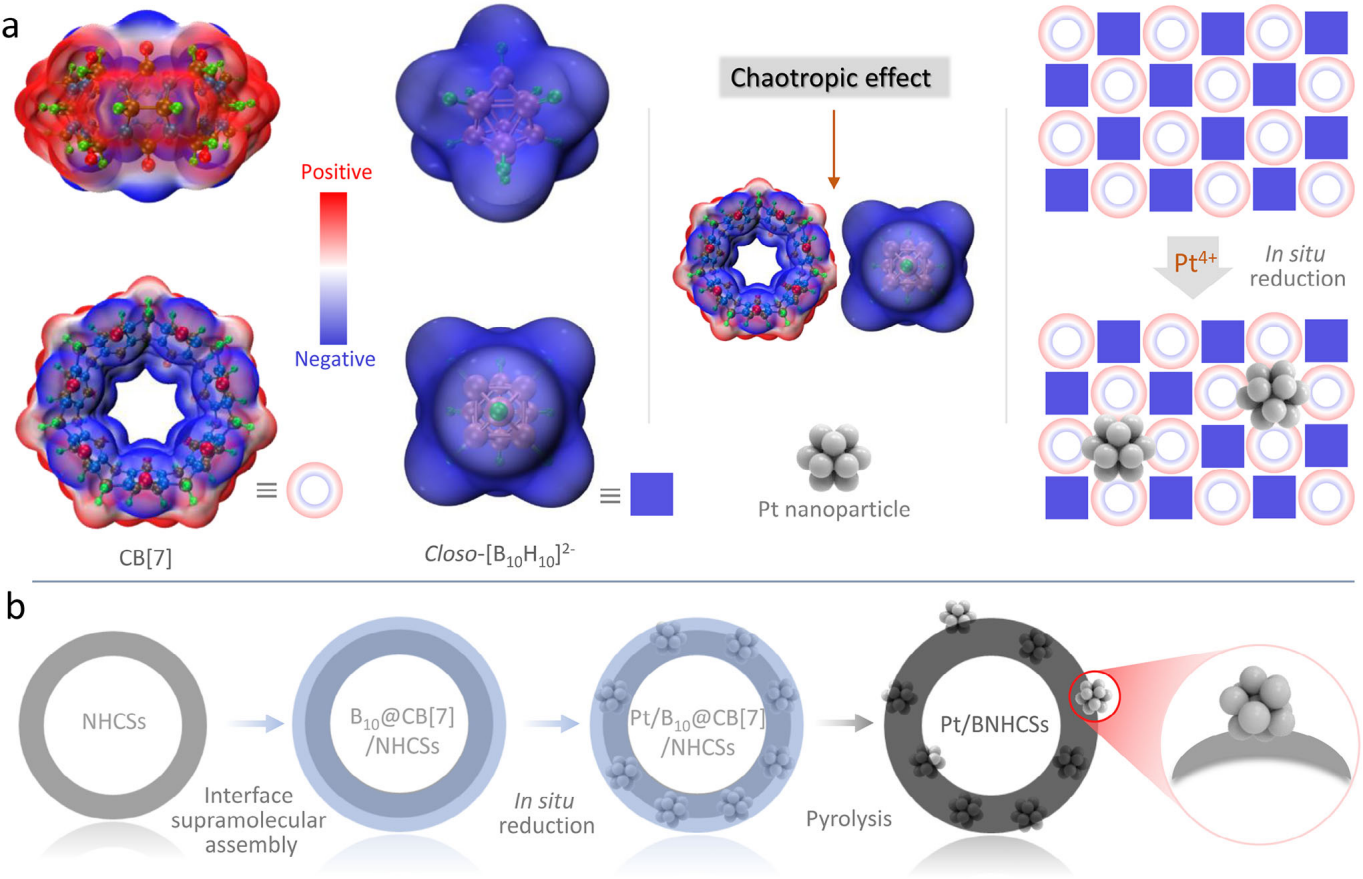
Preparation strategy of Pt/BNCHSs.

**Figure** **1** synthesis scheme of Pt/BNHCSs. Firstly, driven by the electrostatic interaction and the chaotropic effect,^[^ ^10^ ^]^ *closo*‐[B_10_H_10_]^2−^ as the guest quickly self‐assembled into the waist of the host CB[7], and formed a stable supramolecular assembly (named B_10_@CB[7]/NHCSs) on the surface of nitrogen‐doped hollow carbon spheres (NHCSs), so as to achieve the purpose of uniformly dispersing the reducing agent into the carrier. After the addition of Pt^4+^, *closo*‐[B_10_H_10_]^2−^ reduced Pt^4+^ to Pt^0^ in situ. Owing to the reducing agent was confined to the supramolecular framework and was distributed the form of lattice. The Pt obtained by in situ reduction at the boron cluster site was distributed in the NHCSs in a highly dispersed and ultrafine state. Subsequently, the supramolecular organic matrix embedded with nano‐platinum was converted into a boron‐carbon‐nitrogen (BCN) matrix with rich electrical properties by high temperature heat treatment technology to improve the electrocatalytic performance.

The FT‐IR signals clearly shows the formation process of Pt/BNHCSs (Figure S1, Supporting Information). The characteristic signals of *closo*‐[B_10_H_10_]^2−^ and CB[7] appeared at the same time of B_10_@CB[7]/NHCSs, indicating that *closo*‐[B_10_H_10_]^2−^ combined with CB[7]. Additionally, the FT‐IR signal of Pt/B_10_@CB[7]/NHCSs was close to that of B_10_@CB[7]/NHCSs after in situ reduction to construct nano‐platinum, indicating that the supramolecular framework of B_10_@CB[7] was not destroyed, which was related to the reduction of Pt^4+^ by only a small amount of *closo*‐[B_10_H_10_].2^−^ After heat treatment, the FT‐IR signal of Pt/BNHCSs has lost the characteristic signals of *closo*‐[B_10_H_10_]2^−^ and CB[7], and only C═C, C═C, C─N signals exist, indicating that the supramolecular matrix has been graphitized. Element mapping imaging verified that the supramolecular assembly was successfully loaded on the surface of the hollow carbon sphere (Figure S2d–h, Supporting Information), where the B element signal came from the contribution of *closo*‐[B_10_H_10_],2^−^ and the O element corresponded to the contribution of CB[7]. At the same time, B_10_@CB[7]/NHCSs were formed, and the size of the hollow nanospheres increased significantly, which also indicated that the supramolecular coating was successfully coated on the surface of NHCSs.

Compared with B_10_@CB7/NHCSs, Pt with face‐centered cubic (*fcc*) configuration appeared in the powder X‐ray diffraction (PXRD) signal of Pt/B_10_@CB[7]/NHCSs, which confirmed that Pt^4+^ was reduced to Pt^[^ ^11^ ^]^ (Figure S3, Supporting Information). After the heat treatment of Pt/B_10_@CB[7]/NHCSs to form Pt/BNHCSs, the XRD signal peak of Pt was sharper and the half peak width was narrower, indicating that the crystallinity of Pt is enhanced. The existence of Pt^0^ in Pt/BNHCSs was also proved by X‐ray photoelectron spectrometry (XPS), in the XPS signal (Figures S4 and S5, Supporting Information), in addition to the presence of Pt^0^ signal, there is also a small amount of oxidized platinum. Since only the signal of Pt^0^ appears in the XRD results, it indicates that the signal of oxidized platinum in the XPS results comes from the inevitable oxidation of the surface, which is because XPS is a surface analysis method.^[^ ^12^ ^]^ Only zero‐valent platinum signal appears in the TEM signal, which will be discussed later.

**Figure 2:**
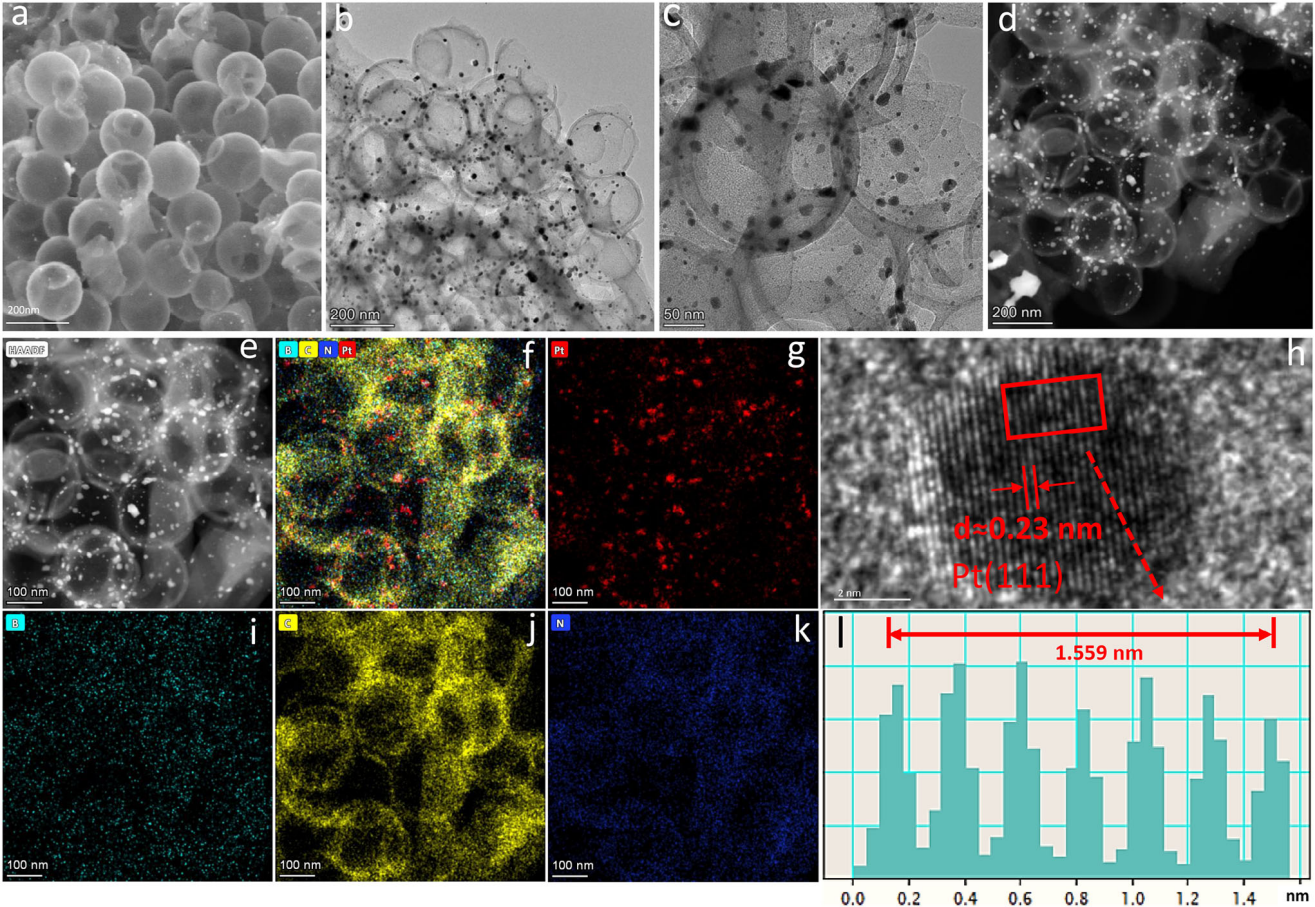
a) SEM image of Pt/BNHCSs, b,c) TEM images of Pt/BNHCSs, d,e) HAADF images of Pt/BNHCSs, f–j) The element distribution in Pt/BNHCSs, k,l) High‐resolution TEM images and lattice spacing of Pt nanoparticles in Pt/BNHCSs.

The morphology and state of Pt/BNHCSs were observed by scanning electron microscopy (SEM) and transmission electron microscopy (TEM) (**Figure** **2**). SEM and TEM images show that the morphology of Pt/BNHCSs is similar to that of NHCSs (Figure 2a; Figure S2a,b, Supporting Information), B_10_@CB[7]/NHCSs (Figure S2c, Supporting Information), and Pt/B_10_@CB[7]/NHCSs (Figure S6, Supporting Information), which is a hollow sphere with good dispersion. After coating the supramolecular to form B_10_@CB[7]/NHCSs, the size of the hollow spheres becomes significantly larger. In the TEM images of Pt/BNHCSs, black nanoparticles with a size of about 10 nm can be clearly observed in the shell structure (Figure 2b–d) (Figure S7, Supporting Information). These black particles have good crystallinity, and the lattice spacing is about 0.23 nm (Figure 2h), which is consistent with the lattice spacing of the Pt(111) crystal plane, so the black nanoparticles shown in the spherical shell are zero‐valent Pt.^[^ ^13^ ^]^ The TEM images further confirm the results of XRD and XPS, and the Pt in Pt/BNHCSs exists in the form of zero valence. The element distribution imaging shows that (Figure 2f,g and i–k), Pt was well dispersed in Pt/BNHCSs, and there are also B, N and C elements in Pt/BNHCSs. These results indicate that the strategy developed in this study to construct a supramolecular framework containing a reducing agent on the surface of NHCSs to construct nano‐metals in situ on the surface of NHCSs is advanced, and is conducive to the construction of highly dispersed and ultrafine nano‐Pt.

**Figure 3:**
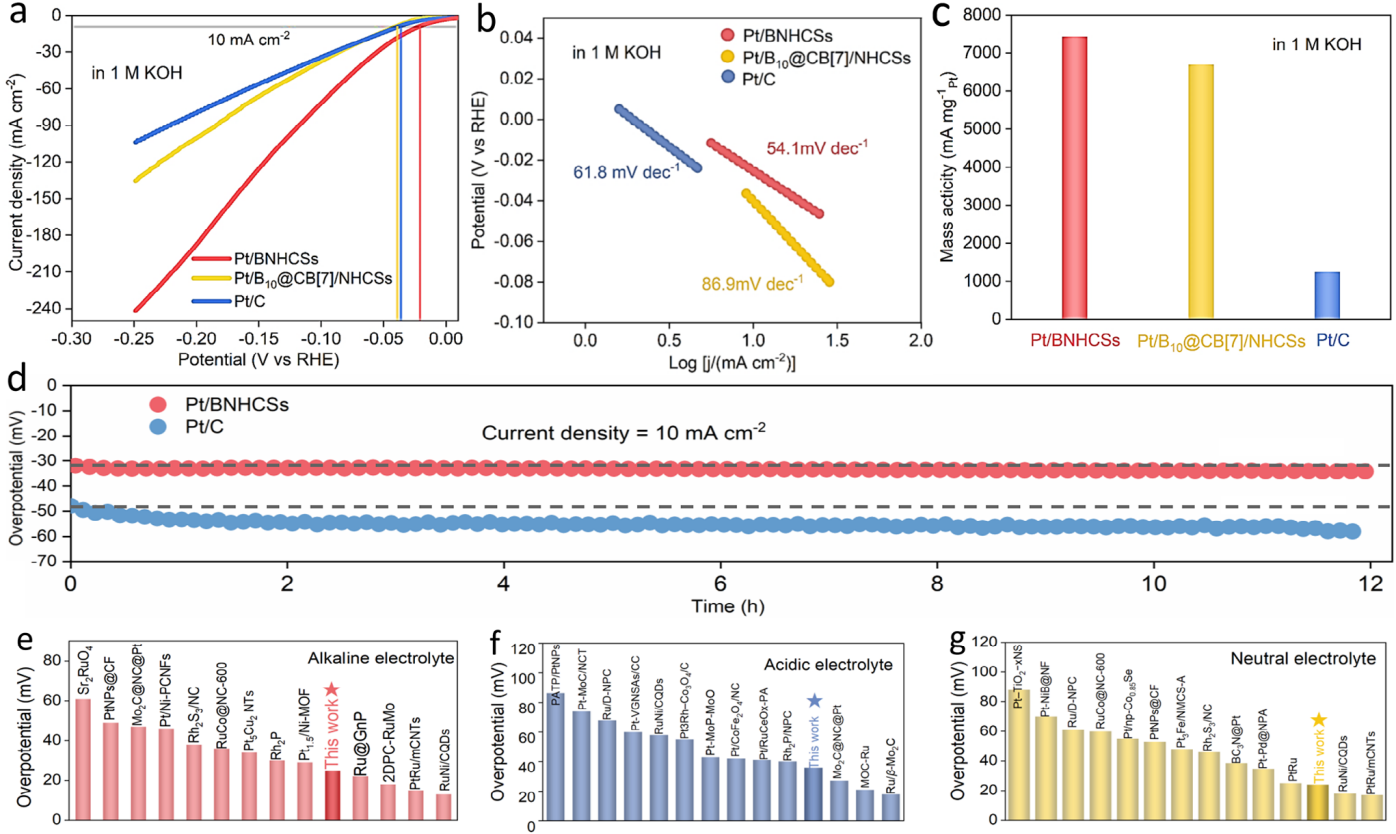
a) The LSV curves of HER catalyzed by Pt/BNHCSs, Pt/B_10_@CB[7]/NHCSs, and Pt/C (20 wt%) in 1 M KOH solution, b) The Tafel slopes of HER catalyzed by Pt/BNHCSs, Pt/B_10_@CB[7]/NHCSs, and Pt/C (20 wt%) in 1 M KOH solution, c) The mass activity of HER catalyzed by Pt/BNHCSs, Pt/B_10_@CB[7]/NHCSs, and Pt/C (20 wt%) in 1 M KOH solution, d) The stability of Pt/BNHCSs and Pt/C (20 wt%) for long‐term catalytic HER in 0.5 M H_2_SO_4_ solution, e–g) The performance of different reported catalysts for HER in alkaline, neutral and acidic media.

As a verification, we explored the performance of Pt/BNHCSs catalytic hydrogen evolution reaction (HER), and further evaluated the effectiveness of the anodic alkaline MOR cathode hydrogen evolution coupling system. In alkaline medium (1 M KOH solution), the overpotential and Tafel slope of Pt/BNHCSs were as low as 24.9 mV and 54.1 mV dec^−1^, respectively, which exceeded the performance of Pt/C (20 wt%) benchmark catalyst. As a fairer comparison, the mass activity of Pt/BNHCSs (mA mg _Pt_^−1^) is close to 7 times that of commercial Pt/C catalyst (**Figure** **3**a–c) when catalyzing alkaline HER. At the same time, the catalytic performance of Pt/BNHCSs for HER in neutral (1 M PBS solution) and acidic media (0.5 M H_2_SO_4_ solution) is also higher than that of commercial Pt/C catalyst (Figure S8, Supporting Information). With Pt/BNHCSs as catalyst, the overpotential and Tafel slope of HER in neutral medium were as low as 24 mV and 38 mV dec^−1^, respectively, and as low as 36 mV and 89 mV dec^−1^ in acidic

medium, respectively (Table S1, Supporting Information). Generally, HER in alkaline media needs to undergo H_2_O dissociation process, so it is more difficult to occur in alkaline media than in acidic media. Here, when Pt/BNHCSs were used as catalysts, although the current signal of HER in acidic medium at high reduction potential is not much different from that of HER in alkaline medium, the overpotential (at 10 mA cm^−2^) of HER in alkaline medium is lower in the potential range where HER occurs at the beginning. This phenomenon indicates that the properties of Pt in Pt/BNHCSs may change greatly compared with pure Pt, which will be discussed later. In addition, it was also observed that after heat treatment of Pt/B_10_@CB[7]/NHCSs to form Pt/BNHCSs, the catalytic activity for HER was greatly improved, regardless of overpotential or Tafel slope. Pt/BNHCSs have the stability of catalyzing HER for a long time under full pH conditions (Figures S9–S11, Supporting Information). When the HER was driven by a current density of 10 mA cm^−2^ in 0.5 M H_2_SO_4_ solution for 12 h, the overpotential of HER catalyzed by Pt/BNHCSs did not decrease significantly, but the overpotential of HER catalyzed by Pt/C increased significantly. Even in acidic electrolyte, Pt/BNHCSs‐catalyzed HER can maintain high performance for a long time, which is beneficial to practical application. At present, the HER performance of Pt/BNHCSs material has been significantly better than most of the reported noble metals catalysts, and has a performance advantage (Figure 3e–g) (Tables S2–S4, Supporting Information).

**Figure 4:**
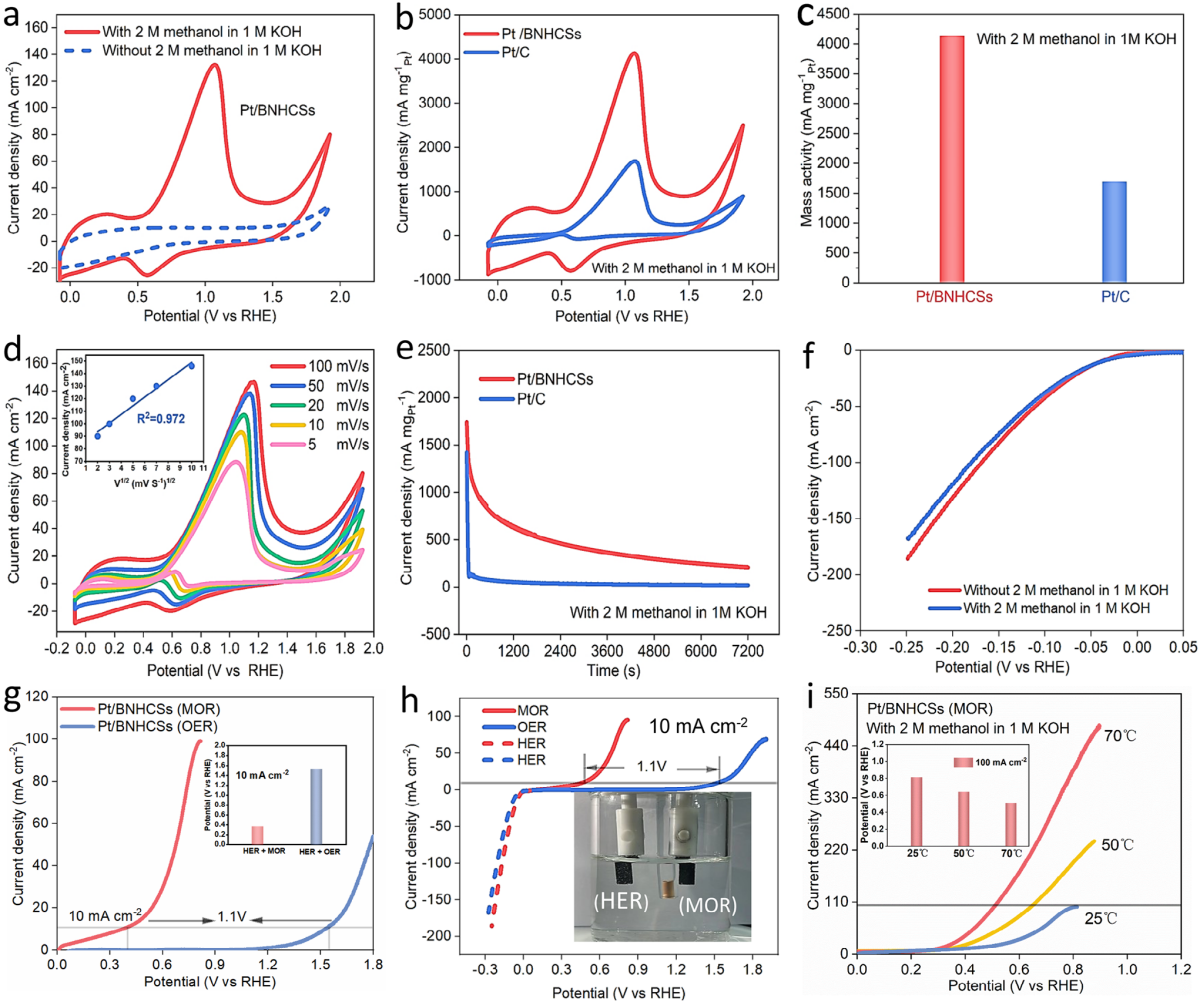
a) The CV curves of MOR catalyzed by Pt/BNHCSs in 1 M KOH solution with and without methanol, b) The CV curves of MOR catalyzed by Pt/BNHCs and commercial Pt/C in 1 M KOH solution containing methanol, c) The mass activity of Pt/BNHCs and commercial Pt/C for MOR (at maximum oxidation current), d) The CV curves of MOR catalyzed by Pt/BNHCSs at different scan rates, e,f) The stability of MOR catalyzed by Pt/BNHCSs and commercial Pt/C, g) The LSV curves of Pt/BNHCSs catalytic MOR and OER in 1 M KOH solution, h) The LSV curves of Pt/BNHCSs when catalyzing HER and MOR coupling system at the same time (red line: electrolyte with methanol, blue line: electrolyte without methanol), i) The LSV curves of MOR catalyzed by Pt/BNHCSs at different temperatures.

To solve the slow kinetics and high cell voltage faced by alkaline HER,^[^ ^14^ ^]^ we evaluated the catalytic performance of Pt/BNHCSs for alkaline methanol oxidation reaction (MOR), which is of great significance for the construction of alkaline fuel cells and the realization of an efficient system for anodic methanol oxidative coupling cathode precipitation. When the electrolyte contains methanol, a strong oxidation peak appears when Pt/BNHCSs was used as a catalyst, indicating that Pt/BNHCSs have the ability to catalyze MOR (**Figure** **4**a). Compared with Pt/B_10_CB[7]/NHCSs (Figure S12, Supporting Information) and commercial Pt/C catalysts, the performance of Pt/BNHCSs for MOR is at least doubled in terms of oxidation current density or mass activity (Figure 4b,c), which is superior to most reported Pt‐based materials (Figure S13, Supporting Information) (Table S5, Supporting Information). When Pt/C was used as the catalyst, the signal of CO reduction appears in the reverse direction of the CV curve when catalyzing MOR,^[^ ^5^, ^15^ ^]^ but there is almost no signal of CO reduction when Pt/BNHCSs was used as the catalyst. This phenomenon may represent that there is no strong adsorption or long‐term aggregation of CO on the surface of Pt/BNHCSs catalyst. By changing the scan rate of the cyclic voltammetry (CV) test, the oxidation signal in the CV curves increases with the increase of the scan rate, and shows a linear correlation (Figure 4d), indicate that the MOR behavior on Pt/BNHCSs is affected by methanol diffusion.^[^ ^16^ ^]^ With the increase of methanol contact frequency, the active sites in Pt/BNHCSs can be fully utilized. At the same time, the stability of Pt/BNHCSs and Pt/C in catalyzing MOR was also compared. The stability of Pt/BNHCSs was significantly better in long‐term operation (Figure 4e) or CV test for 1000 times (Figure S14, Supporting Information).

**Figure 5:**
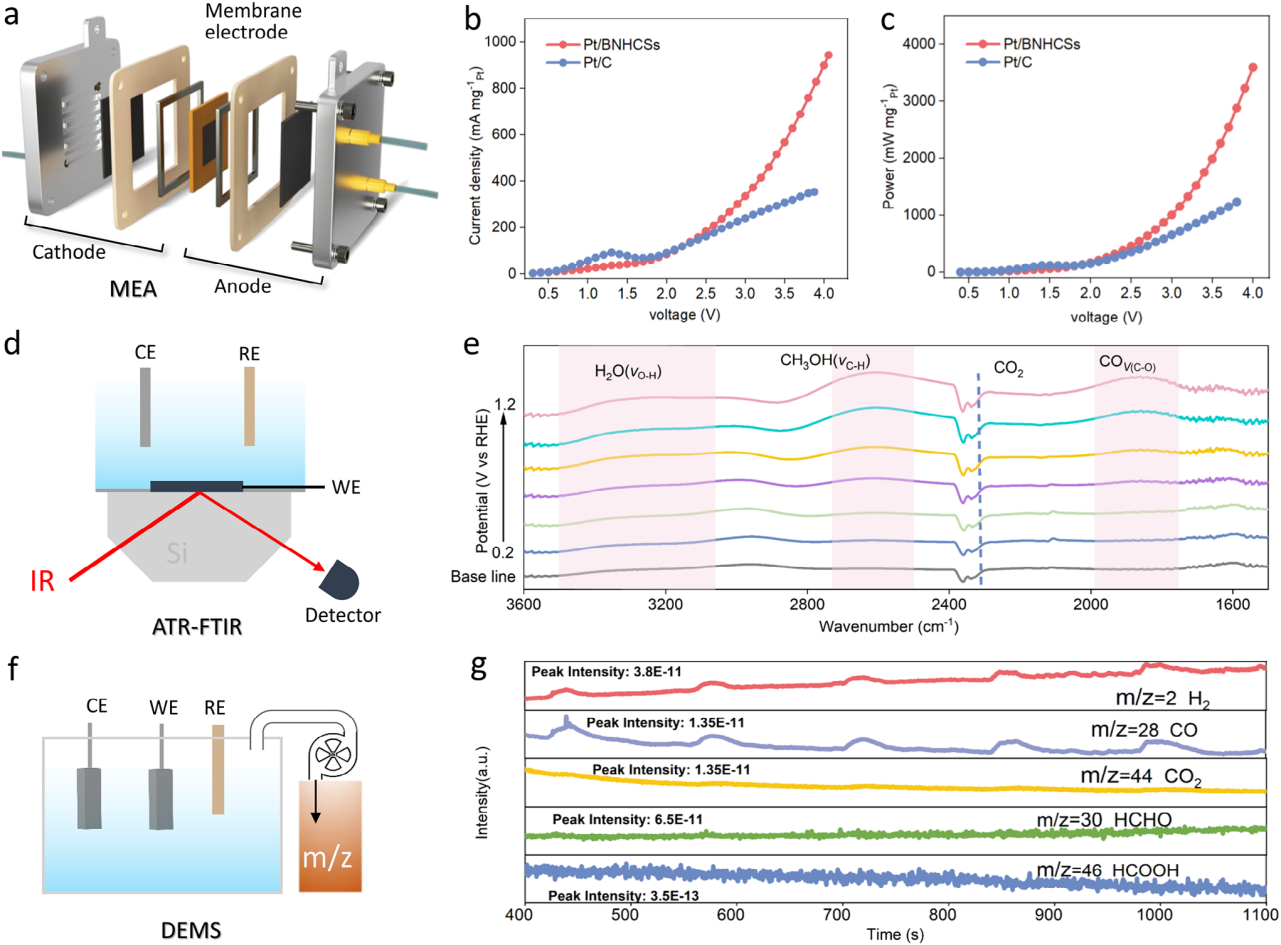
a) The composition of the electrolytic cell of the membrane electrode assembly (MEA), b,c) The LSV curves of Pt/BNHCSs and commercial Pt/C in MEA when simultaneously catalyzing HER and MOR, b is mass normalized activity, c is power density, d) In situ electrochemical ATR‐FTIR test principle, e) The in situ electrochemical ATR‐FTIR signals when Pt/BNHCSs catalyzed MOR, f) *On‐line* electrochemical DEMS test principle, g) The *on‐line* electrochemical DEMS signals when Pt/BNHCSs catalyzed MOR and HER.

Furthermore, the performance of Pt/BNHCSs for simultaneous cathodic HER and anodic MOR was evaluated. On the one hand, the introduction of methanol into the electrolyte did not significantly affect the performance of the cathode HER (Figure 4f). However, the anode current density was greatly improved, and the overpotential required to achieve a current density of 10 mA cm^−2^ was reduced by at least 1.1 V (Figure 4g,h), which is superior to most of the reported results (Figure S15, Supporting Information) (Table S6, Supporting Information), this result shows that Pt/BNHCSs can be used as a catalyst to effectively construct a cathode hydrogen evolution and anode alkaline methanol oxidation system, which can effectively reduce the cell voltage when electrolysis of water to produce hydrogen, which is conducive to improving energy utilization efficiency. It was also observed that Pt/BNHCSs could promote the electrochemical oxidation of methanol at different temperatures, and the oxidation performance increased with the increase of temperature without poisoning (Figure 4i). In addition to the excellent electrochemical stability (performance), the structure of Pt/BNHCSs after simultaneous catalytic cathodic HER and anodic MOR was similar to that of the initial state (Figure S16, Supporting Information). Pt still exists in the form of zero valence in Pt/BNHCS and remains in the *fcc* configuration. When Pt/BNHCSs or commercial Pt/C were assembled on the anion exchange membrane to form a membrane electrode assembly (MEA) (Figure 5a–c; Figure S17, Supporting Information), the MEA based on Pt/BNHCSs has a lower cell voltage at the same current density. Note, when Pt/C was employed as the anode catalyst in the MEA, the signal observed around 1.2 V in the LSV curve originates from the oxidation of CO, which is generated during the methanol oxidation process, on the Pt/C catalyst surface. All these indicate that the Pt/BNHCSs developed in this study are conducive to the construction of a cathode HER‐coupled anode MOR high‐efficiency electrolysis system.

In situ electrochemical attenuated total reflection Fourier transform infrared (ATR‐FTIR) spectroscopy and on‐line differential electrochemical mass spectrometry (DEMS) were used to monitor the reaction behavior and product distribution at the electrode interface. In the ATR‐FTIR signals, when HER occurs at the Pt/BNHCSs interface (Figure S18, Supporting Information), as the reduction potential increases, the adsorption capacity of the catalyst interface to H_2_O gradually increases, which is reflected in the continuous increase of H_2_O signal intensity. In alkaline medium, the adsorption of H_2_O on the catalyst determines whether HER can achieve a high reaction rate.^[^ ^17^ ^]^ Obviously, Pt/BNHCSs can realize the adsorption, conversion and re‐adsorption of H_2_O at the reduction potential. When MOR (Figure 5d,e) occurs at the Pt/BNHCSs interface, the enhanced adsorption of methanol and H_2_O molecules was also observed with the increase of reduction potential, which plays an important role in providing reactive oxygen species. At the same time, it was observed that the CO signals at the Pt/BNHCSs interface gradually increased, while the CO_2_ signals was not significantly enhanced. Therefore, the process of MOR on Pt/BNHCSs is more inclined to CH_3_OH → CO, rather than CH_3_OH → CO → CO_2_. In the online DEMS test, when Pt/BNHCSs were used as both cathode catalyst and anode catalyst, it was observed that H_2_ and CO signals were obviously generated in the electrolysis system, and only weak CO_2_ signals were generated. In addition, no signals of formaldehyde (HCHO) and formic acid (HCOOH) were observed in the online DEMS results (Figure 5f,g). The online DEMS results were consistent with the monitoring results of in situ electrochemical ATR‐FTIR, which effectively reveals the evolution process of the interface reaction, and also provides important evidence for further verification of the catalytic mechanism by density functional theory calculations (DFT).

**Figure 6:**
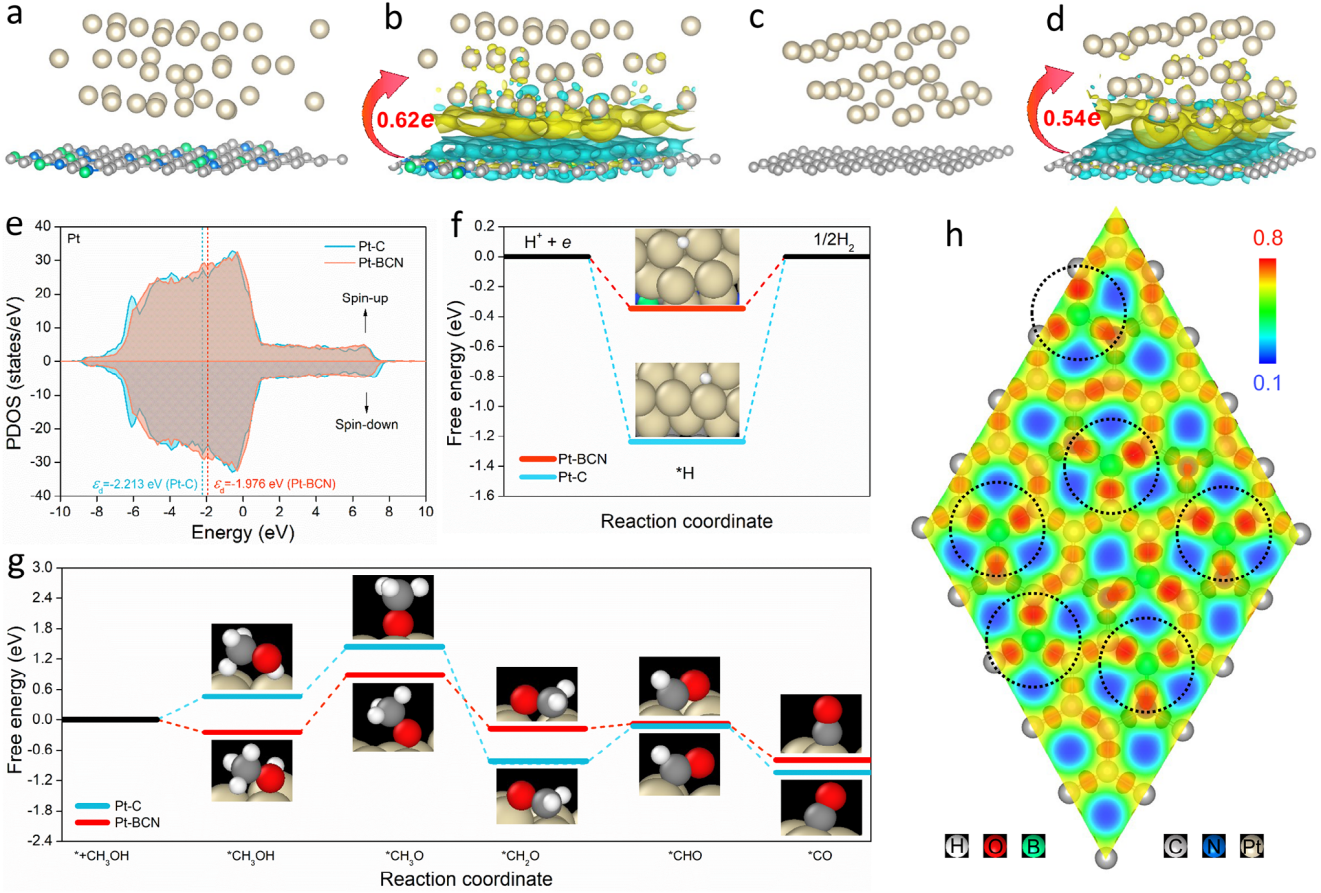
a) Pt‐BCN model, b) Differential charge distribution of Pt‐BCN, c) Pt‐C model, d) Differential charge distribution of Pt‐C, e) Projected density of state (PDOS) Pt in Pt‐C and Pt‐BCN, f) The free energy step diagram of HER on Pt‐BCN and Pt‐C, g) The free energy step diagram of MOR on Pt‐BCN and Pt‐C, h) Electron localization function of BCN.

The reductant (boron cluster)‐preset supramolecular strategy not only provides favorable conditions for the construction of highly dispersed nano‐Pt but also introduces a BCN support rich in heteroatoms. Consequently, Pt‐BCN model (Pt placed on BCN) (**Figure** **6**a) and Pt‐C model (Pt placed on graphite carbon) (Figure 6c) were separately constructed to investigate the effects of the surrounding environment of Pt. Differential charge distribution analysis reveals that, compared to Pt‐C, Pt in Pt‐BCN can acquire more electrons from the support (Figure 6b,d), altering the position of the *d*‐band center (*ɛ*_d_) (Figure 6e), which facilitates the activation of Pt. The adsorption capacity of Pt on Pt‐BCN for active hydrogen (H) is significantly weaker than that of Pt on Pt‐C (Figure 6f), which makes the former more favorable for the formation and desorption of H_2_. Unlike HER in acidic media, HER in alkaline media usually undergoes the dissociation process of H_2_O. When *H_2_O was converted to *H and *OH at the Pt sites on the Pt‐BCN, the dissociation energy is ‐0.171 eV, while the dissociation energy at the Pt sites on the Pt‐C is 0.535 eV (Figure S19, Supporting Information). The more negative H_2_O dissociation energy indicates that Pt anchored on BCN is more conducive to promoting alkaline HER. Since the dissociation energy of H_2_O on Pt‐BCN is negative, indicating that the dissociation of H_2_O tends to occur spontaneously, the dissociation process of H_2_O may not be the main obstacle to the occurrence of HER on Pt‐BCN, which may be the reason why the η_10_ of HER in alkaline medium is lower than that in acidic medium at a smaller reduction potential. In situ electrochemical ATR‐FTIR and online DEMS test results, no obvious CO_2_, HCHO and HCOOH were observed on the electrode surface, and only obvious CO products were detected, and CO was the most oxidized product in this study. Since no HCOOH product (or intermediate) was detected, there was no obvious oxygen insertion oxidation process. Based on the experimental phenomena and previous reference reports,^[^ ^1^, ^3^, ^18^ ^]^ we speculate that the MOR at the Pt site on Pt/BCN may be carried out in the form of stepwise dehydrogenation oxidation (Equation.R1–4 shown in Supporting information). In MOR, the adsorption of CH_3_OH on Pt‐BCN is spontaneous, whereas adsorption on Pt‐C requires overcoming an energy barrier (Figure 6g). Furthermore, after the formation of Pt‐BCN, the conversion from *CH_2_O to *CHO proceeds more smoothly.

Experimentally, when BNHCSs without Pt loading were used as catalysts alone, there was almost no catalytic activity (Figures S20 and S21, Supporting Information), but DFT calculations confirmed the effective regulation of Pt properties by the support. The different effects of graphite carbon and BCN as supports on the properties of Pt arise from the differences in electronegativity among B, C, and N in BCN.^[^ ^19^ ^]^ The BCN support tends to form local dipoles (Figure 6h), which is conducive to promoting the transfer of electrons. Electrochemical impedance spectroscopy (EIS) confirmed the speculation that BNHCSs as a carrier can reduce the electron transfer resistance^[^ ^20^ ^]^ (Figure S22, Supporting Information). In addition, due to the introduction of hollow carbon matrix as a carrier, and the introduction of interfacial supramolecular coating and in situ reduction strategy, the dispersion of Pt sites was greatly improved and the size of Pt was reduced. Therefore, it is observed that the electrochemical surface active (ECSA) region of Pt/BNHCSs is much higher than that of commercial platinum carbon ECSA (Figure S23, Supporting Information). These are all factors that cause Pt/BNHCSs to have both catalytic electrochemical hydrogen evolution reaction and alkaline methanol oxidation reaction performance. In summary, this study developed a strategy of boron cluster reductant preset into supramolecular to realize the in situ controllable construction of nano‐Pt, introduced Pt into the BCN matrix with rich electrical properties with hollow carbon‐supported, realized multi‐scenario electrolysis of water to produce hydrogen and constructed a high‐performance cathodic hydrogen evolution‐anodic methanol oxidation coupling system, showing a future ecology of low‐energy green energy production.

## 2 Conclusions

In this work, an innovative strategy was developed to directly introduce the reducing agent boron cluster into the framework to construct a reductive supramolecular by constructing a supramolecular combination of boron clusters and cucurbituril[7] (CB[7]). The reductive supramolecular can effectively promote the in situ conversion of Pt^4+^ to highly dispersed small‐sized nano‐platinum, and build active functional sites on the boron‐carbon‐nitrogen (BCN) matrix, creating a high‐performance bifunctional catalyst (Pt/BNHCSs). Pt/BNHCSs can promote electrocatalytic water to produce hydrogen under various conditions, and the overpotentials (η_10_) in alkaline, neutral and acidic media are as low as 24.9, 24.0 and 36 mV, respectively, and show lasting stability. Pt/BNHCSs can also accelerate the kinetics of anodic alkaline methanol oxidation, which is superior to commercial Pt/C (20 wt%) catalyst in all aspects. The cathodic hydrogen evolution‐anodic methanol oxidation coupling system constructed using the Pt/BNHCSs greatly reduced the overall energy consumption of the electrolysis system. In situ ATR‐FTIR and online DEMS revealed that the catalyst interface enhanced H₂O adsorption and promoted the CH₃OH→CO conversion process, and density functional theory calculations indicated that the BCN support facilitated the evolution of H₂O to H₂ and CH₃OH to CO, elucidating the mechanism by which Pt/BNHCSs simultaneously promoted hydrogen production and methanol oxidation.

## Author Contributions

R.B. and Q.Y. contributed equally to this work. X.Z., Y.Z., Y.Z., and Y.Z. conceived this idea and designed this study. R.B. prepared various catalysts and carried out experiments. X.Z. and Y.Z. jointly constructed the theoretical calculation model and implemented the density functional theory calculations. X.Z., Y.Z., X.Z., Q.Y., C.L., and H.W. participated in the characterization of the catalysts X.Z. and R.B. wrote the manuscript. X.Z., Y.Z., Y.Z., and Y.Z. reviewed and revised the manuscript. X.Z. provided financial support.

## Conflict of Interest

The authors declare no conflict of interest.

## Supporting information



Supporting Information

## Data Availability

The data that support the findings of this study are available from the corresponding author upon reasonable request.
