# Supplementary material for: Reductive Supramolecular In Situ Construction of Nano‐Platinum Effectively Couples Cathodic Hydrogen Evolution and Anodic Alcohol Oxidation
Source: Adv Sci (Weinh). 2025 Apr 3;12(26):2502002. doi: 10.1002/advs.202502002 (PMC12245136; doi:10.1002/advs.202502002)
Supplement: Supplementary file 1 — Supporting Information [file ADVS-12-2502002-s001.docx]

Supporting Information for

**Reductive Supramolecular *in-situ* Construction of Nano-Platinum Effectively Couples Cathodic Hydrogen Evolution and Anodic Alcohol Oxidation**

**Supplementary text**

**1. Experimental**

**1.1 Materials and Chemical**

Ammonia (25%), hydrofluoric acid (40%) and anhydrous ethanol (95%) were purchased from Tianjin Zhiyuan Chemical Reagent Co., Ltd.；Ethyl orthosilicate (28%) was purchased from Tianjin Damao Chemical Reagent Factory, Dopamine hydrochloride (98%) was purchased from Aladdin Chemical Reagent Co., Ltd.；Tris (99%) was purchased from Shanghai Yien Chemical Technology Co., Ltd. H_2_PtCl_6_ (99%) was purchased from Shanghai Macklin Biochemical Technology Co., Ltd. (Et_3_H)_2_[B_10_H_10_] (98%) was purchased from Zhengzhou Ruke Biological Co., Ltd.and Na_2_[B_10_H_10_] was obtained by ion exchange；CB[7] was synthesized according to the method of literature^[1]^；KH_2_PO_4_ (99%) and K_2_HPO_4_ (99%) were purchased from Tianjin Zhiyuan Chemical Reagent Co., Ltd.；KOH (95%) is located in Shanghai Yien Chemical Technology Co., Ltd., and methanol (99%) is purchased from Chengdu Cologne Chemical Co., Ltd. ; H_2_SO_4_ (98%) was purchased from Yunnan Yanglin Industrial Development Zone Shandian Pharmaceutical Co., Ltd., and 20 wt% Pt/C was purchased from Shanghai McLin Biochemical Technology Co., Ltd.

**1.2 Preparation of nitrogen-doped hollow carbon spheres (NHCSs)**

At room temperature, 400 mL of anhydrous ethanol was mixed with 200 mL of ultrapure water and 30 mL of 25% ammonia solution was added. After stirring at 1000 rpm for 10 min, 30 mL of tetraethyl orthosilicate solution (added completely in 5 min) was added and stirred at 1000 rpm for 1 h. Then the obtained white precipitate is centrifuged (Centrifugation rate 9000 rpm, time 5 min). The solid obtained by centrifugation was washed twice with ultrapure water, and the collected white solid was dissolved in 400 mL Tris-HCl (60.5 g Tris dissolved in 1 L of water, adjusted to pH=9 with 1 M HCl) solution. Add 50 mL dopamine hydrochloride solution (200 g L^-1^), stirring at room temperature for 24 h, and vacuum filtration, then ethyl alcohol washing 3 times, drying at 80 °C in vacuum drying oven. The dried materials were calcined in a tube furnace under N_2_ atmosphere. The parameters of the tube furnace were (programmed to 700 °C, heating rate of 5 °C/min, holding time of 2 h). 1 g material corresponded to 10 mL 20% HF solution, and etching was performed in a polytetrafluoroethylene beaker for 24 h. Then a large amount of water was added to dilute it so that its pH was close to 7. The solid was transferred to a 250 mL volumetric flask, and the hollow carbon spheres (NHCSs) dispersed in the solution were obtained by constant volume with ultrapure water.

**1.3 Preparation of cucurbituril CB[7] and *closo*-[B_10_H_10_]^2-^ supramolecular self-assembly on NHCSs (B_10_@CB[7]/NHCSs)**

After ultrasonic dispersion for 30 min, CB[7] solution (1 mmol CB[7] dissolved in 100 mL water ) was added and stirred at 700 rpm for 30 min. Na_2_B_10_H_10_ solution (1 mmol Na_2_B_10_H_10_ dissolved in 50 mL water) was added to the above mixture within 5 s. After stirring for 2 h, the solid was collected by vacuum filtration. After washing with pure water for 3 times, the solid was ultrasonically dispersed in 150 mL water to obtain B_10_@CB[7]/NHCSs.

**1.4 Preparation of Pt/BNHCSs**

The above-mentioned dispersed suspension was transferred to a round-bottom flask, and 51.8 mL 7.723 mmol L^-1^ H_2_PtCl_6_ solution was added after the oil bath was kept at 100 °C for 10 min. The mixture was stirred in the oil bath for 2 h. After the mixture was cooled, the solid was collected by vacuum filtration. After washing with pure water, the solid was dried in a blast oven at 80 °C for 6 h to obtain Pt/B_10_@CB[7]/NHCSs. Pt/BNHCSs were prepared by high temperature pyrolysis using Pt/B_10_@CB[7]/NHCSs as raw materials. Pt/BNHCSs were calcined at high temperature in a tube furnace under the protection of N_2_ atmosphere. The parameters of the tube furnace were as follows: 700 °C, heating rate of 5 °C/min, holding time of 2 h, and natural cooling to room temperature to obtain Pt/BNHCSs.

**2.Material characterization method**

The microstructure of the material was observed by scanning electron microscopy (SEM, ZEISS) and transmission electron microscopy (JEOL JEM-F200). Among them, the material is directly pasted on the conductive adhesive to collect SEM imaging. The signal acquisition mode of SEM is InLens, and the acceleration voltage is 3.00 KV; The material was ultrasonically dispersed in ethanol solution, and then dropped on an ultra-thin micro-grid. After natural drying, TEM images were collected on TEM. The XRD signal of the material was collected on an X-ray diffractometer (XRD, Miniflex600). The XPS signal of the material was collected on the X-ray photoelectron spectrometer (XPS, Thermo Kalpha).

**3. Electrochemical test**

**3.1 HER（Hydrogen Evolution Reaction）**

All hydrogen reduction reactions (HER) are carried out at room temperature (25°C) and atmospheric pressure (Yunnan, China). CHI660E electrochemical workstation was used. The H-type electrolytic cell with three-electrode system was used. The two cathodes and anodes of the electrolytic cell were bounded by proton exchange membrane (Nafion 117). The reference electrode and working electrode were used in the cathode chamber, and the counter electrode was used in the anode chamber. In this work, Pt wire was used as the counter electrode, and carbon paper coated with Pt/BNHCSs ink (CP, effective geometric area of about 0.5 cm^2^) was used as the working electrode. 10 mg Pt/BNHCSs were weighed, 10 μL Nafion (5%) was used as adhesive, 640 μL isopropanol and 350 μL ultrapure water were used as dispersant, and the ink with uniform distribution of Pt/BNHCSs was formed by ultrasonic treatment for 20 min. When the electrolyte was 1 M KOH (pH=14) solution, the reference electrode was a Hg/HgO electrode filled with 1 M KOH, and the electrode potential was 0.098 V vs RHE. When 0.5 M PBS (pH=7) solution was selected, the reference electrode was an Ag/AgCl electrode filled with saturated KCl, and the electrode potential was 0.197 V vs RHE. When 0.5 M H_2_SO_4_ (pH=0) was selected, the reference electrode was an Ag/AgCl electrode filled with saturated KCl, and the electrode potential was 0.197 V vs RHE. All electrolytes were bubbling with high-purity Ar gas (99.99%) for 15 min before testing. The LSV curve was scanned at a rate of 10 mV s^-1^, and the results were subjected to 95% IR compensation. Before each scan, the cyclic voltammetry (CV) program was selected to activate the working electrode, and the impurities on the electrode surface were removed to improve its catalytic activity.

**3.2 Methanol oxidation (MOR)**

Similarly, the above three-electrode system was used. The Pt wire was used as the counter electrode, and the carbon paper coated with Pt/BNHCSs ink (CP, effective geometric area of about 0.5 cm^2^) was used as the working electrode. The electrolyte was 1 M KOH solution containing 2 M methanol. The reference electrode was a Hg/HgO electrode filled with 1 M KOH, and the electrode potential was 0.098 V vs RHE. Before the cyclic voltammetry test, the electrolyte was purged with high-purity Ar gas for 15 min, and the working electrode was activated in 1 M KOH solution. After 30 cycles, the CV curve reached a stable state. The CV curve obtained (with Ar bubbling electrolyte for 1 h) by scanning two cycles at 50 mV s^-1^ was used to calculate ECSA. The calculation formula is as follows:

ECSA =$\frac{Q_{H}}{0.21\times\nu\times m_{Pt}}$

Among them, *Q*_H_ represents the peak area charge of hydrogen desorption (m*C* cm^-2^), 0.21 represents the hydrogen evolution constant of Pt (mC cm^-2^), *v* represents the scan rate (mV s^-1^) of CV, and *m*_Pt_ represent the loading of metal Pt on carbon paper in the catalyst (g m^-2^).

The activated carbon paper was used for MOR performance test, and the CV curve scanning rate was 50 mV s^-1^. In the impedance test, the electrolyte was 2.5 mmol L^-1^ potassium ferricyanide solution containing 0.1 M KCl solution.

**4. *In-situ* ATR-FTIR test**

*In-situ* attenuated total reflection Fourier transform infrared (ATR-FTIR) was performed using an infrared spectrometer (ThermoFisher NIcolet IS20) (**Figure S24**). The 20 mg Pt/BNHCSs was dissolved in 2 mL ultrapure water and sonicated for 30 minutes before the reaction. The catalyst was uniformly coated on the gold-plated silicon crystal as a working electrode. The ATR-FTIR signals of HER was collected in 1 M KOH solution, and the ATR-FTIR signal of MOR was collected in 1 M KOH solution containing 2 M methanol. The signal acquisition range is 4000~1000 cm^-1^, the potential range of the infrared signal of HER is 0~-0.5 V, and the potential range of the MOR signal acquisition is 0.2~1.2 V. Before collecting all signals, background acquisition is performed at an open circuit voltage.

**5. On-line differential electrochemical mass spectrometry (DEMS)**

First, the ink is dripped onto a gold-plated glassy carbon electrode with a pipette gun, about 20 μL. After the solvent is completely volatilized, the ink-coated electrode (as the working electrode) is installed in a closed three-electrode electrolytic cell with a gas channel. The counter electrode is a platinum wire electrode, and the reference electrode is a silver chloride reference electrode filled with saturated potassium chloride solution. The electrolyte was 1 M KOH solution containing 2 M methanol, and the electrochemical workstation for collecting signals was Bio-Logical. Before the test, the electrolysis system was allowed to stand for one minute, and the electrochemical signal was collected after the entire system was stabilized. The current applied during the constant current test is −20 mA cm^−2^, with each current applied for 20 s, and then rested for 310 s to complete a test cycle. The gas at the upper end of the electrolyte was collected and qualitatively analyzed by online differential electrochemical mass spectrometry (DEMS).

**6. Density functional theory (DFT) calculation**

The density functional theory calculations were performed based on the Vienna *Ab initio* simulation package (VASP), and the projected augmented wave (PAW) method was used to describe the interaction between valence electrons and ion nuclei, and the generalized gradient approximation (GGA) with Perdew-Burke-Ernzerhof (PBE) functional was used to describe the electron exchange correlation energy.^[^[^1^](#_ENREF_3_1)^]^ The cutoff energy was set to 400 eV, and the convergence threshold for energy was set at 10^-4^ eV. Based on the XRD and high-resolution TEM results, we constructed Pt(111) nanoparticles supported on graphite carbon and Pt(111) nanoparticles supported on boron-carbon-nitrogen (BCN) network, named Pt-C and Pt-BCN, respectively. Some thermodynamic parameters are directly used in the DFT calculation, such as the Gibbs free energy of 14.22 eV for H_2_O and 6.8 eV for H_2_.^[^[^2^](#_ENREF_3_5)^]^ The Gibbs free energy change was calculated by the calculated hydrogen electrode (CHE) model proposed by Norskov et al.^[^[^3^](#_ENREF_3_6)^]^, the calculation method was as follows.

$$\Delta G=\Delta E+\Delta E_{ZPE}-T\Delta S$$

Here $\Delta G$ represents the Gibbs free energy change of the reaction, $\Delta E$ represents the energy change of the HER intermediate before and after adsorption on the catalyst model, $E_{ZPE}$ represents the zero-point energy, *T* is the temperature (298.15 K), $S$ is the system entropy.

The elementary reactions of methanol oxidation reaction (MOR) determined in this study were as follows:

R1: $*CH_{3}OH+OH^{-}\to*CH_{3}O+e^{-}+H_{2}O$

R2: $*CH_{3}O+OH^{-}\to*CH_{2}O+e^{-}+H_{2}O$

R3: $*CH_{2}O+OH^{-}\to*CHO+e^{-}+H_{2}O$

R4: $*CHO+OH^{-}\to*CO+e^{-}+H_{2}O$

**Supplementary Figures and Tables**


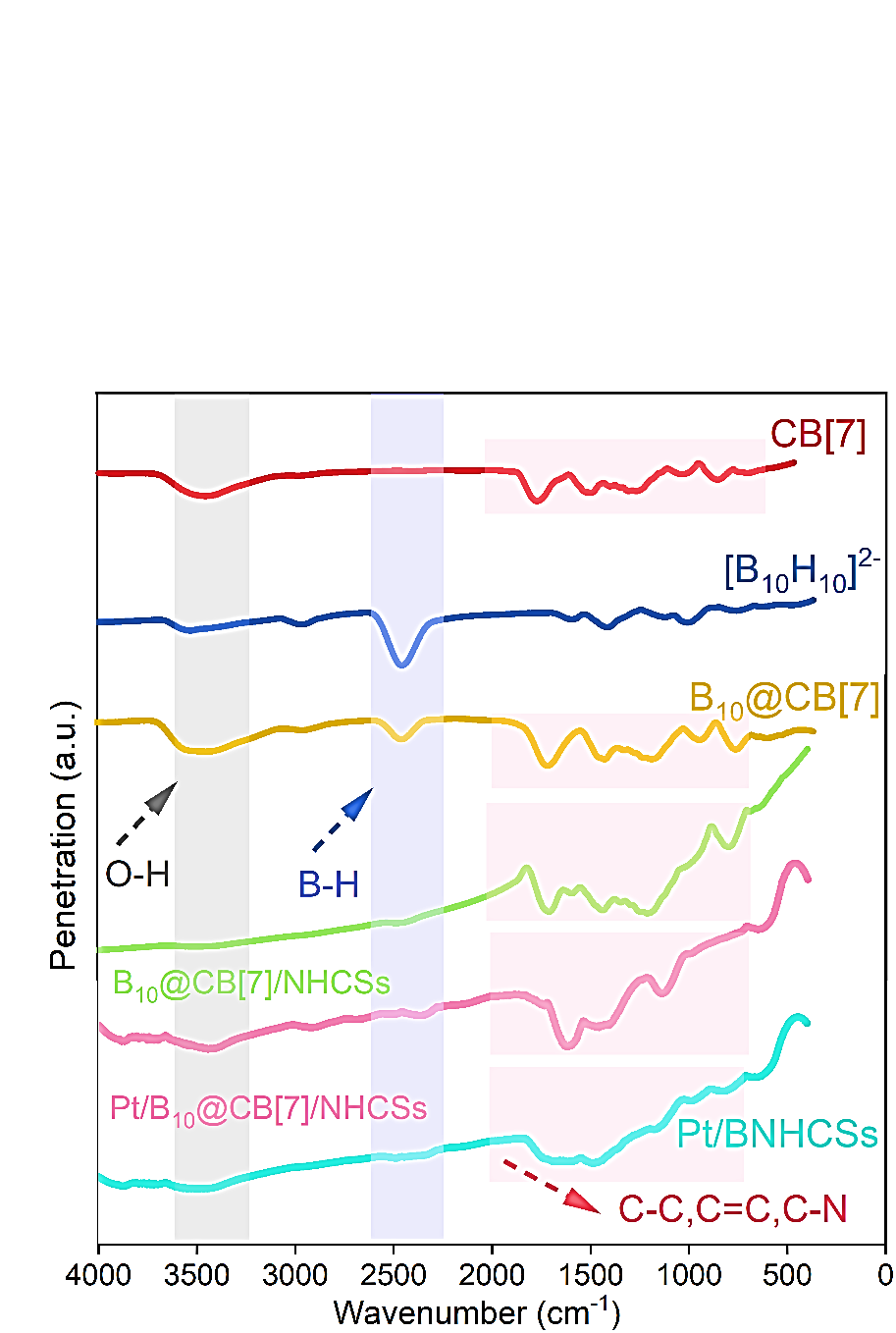


**Figure S1**. FT-IR spectra of CB[7], *closo*-[B_10_H_10_]^2-^, B_10_@CB[7], B_10_@CB [7]/NHCSs, Pt/B_10_@CB[7]/NHCSs, Pt/BNHCSs.
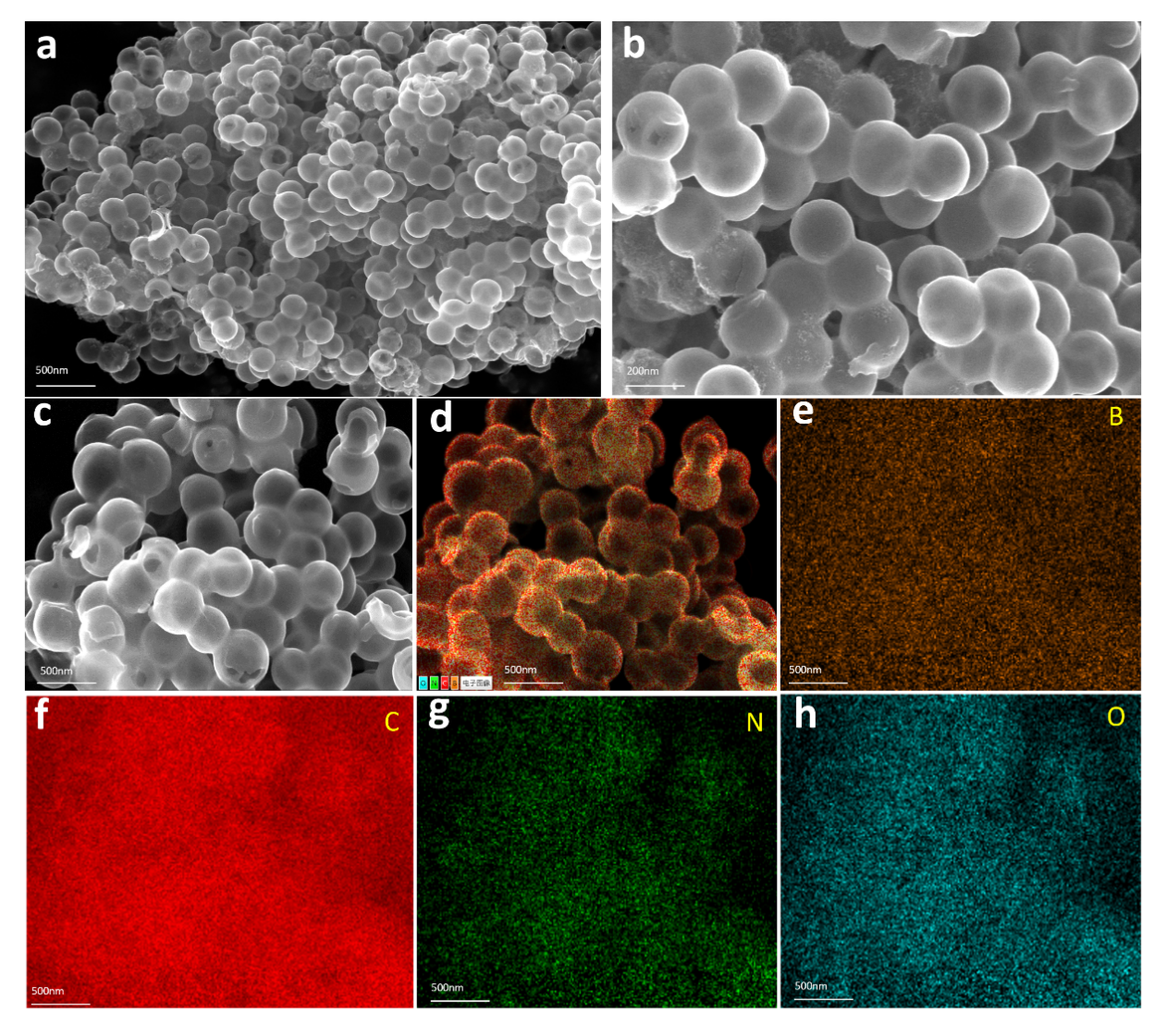


**Figure S2.** (a-b) SEM images of NHCSs; (c) SEM images of B_10_@ CB[7]/NHCSs; (d-h) element mapping of B_10_@CB[7]/NHCSs.


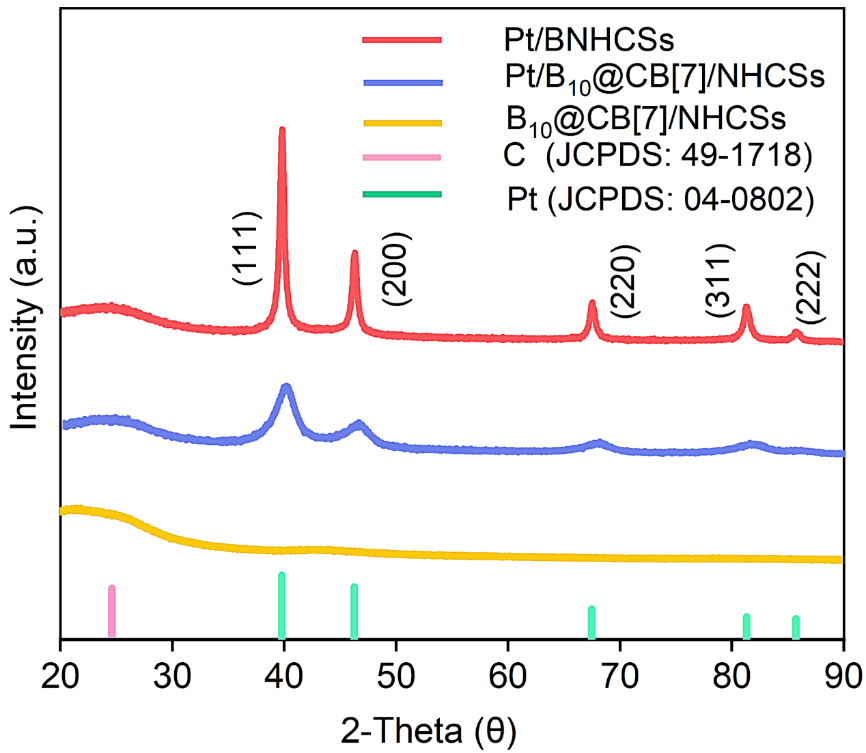


**Figure S3.**PXRD of Pt/BNHCSs,Pt/B_10_CB[7]NHCSs,B_10_@CB[7]/NHCSs.


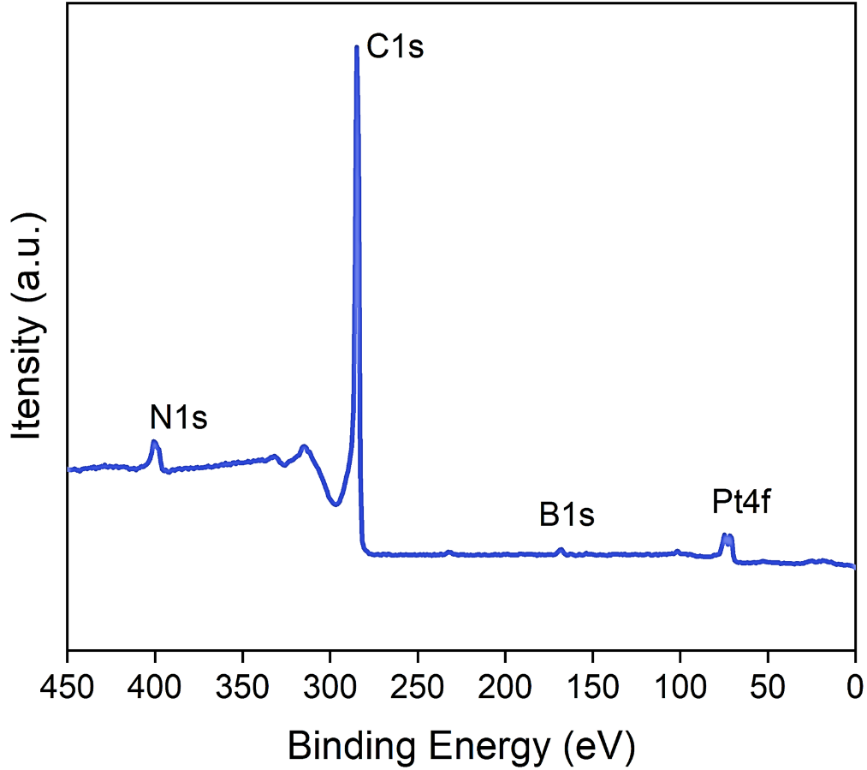


**Figure S4**.Pt/BNHCSs XPS elemental full spectrum.


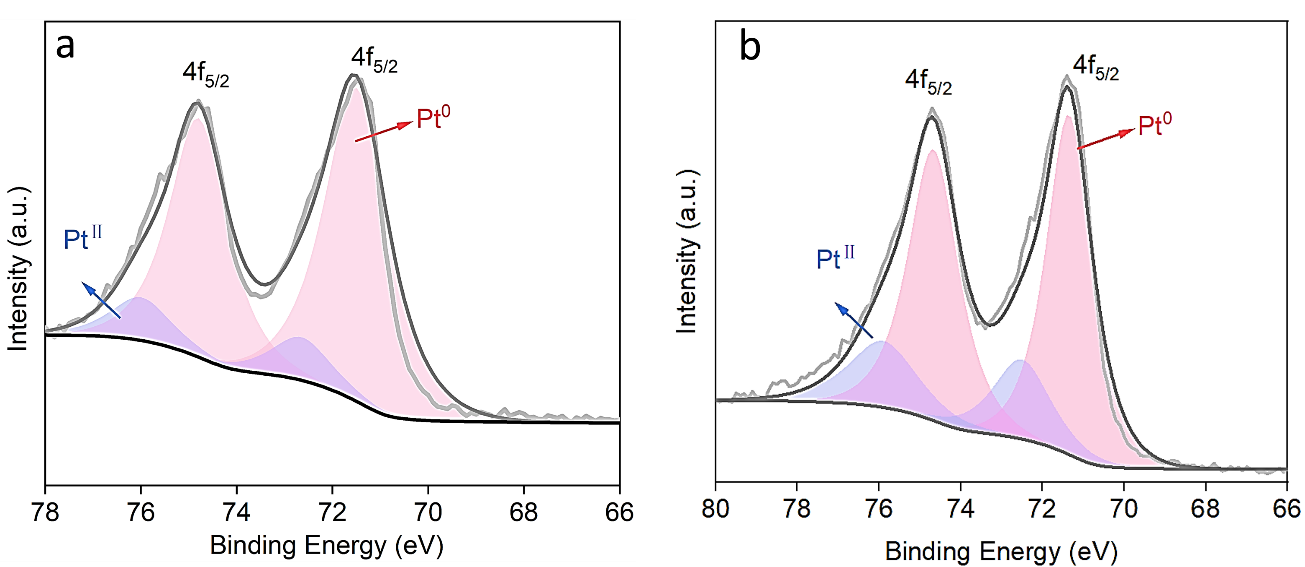
 **Figure S5.** (a) XPS spectra of Pt/BNHCSs; (b) XPS spectra of Pt/B_10_@CB[7] /NHCSs.


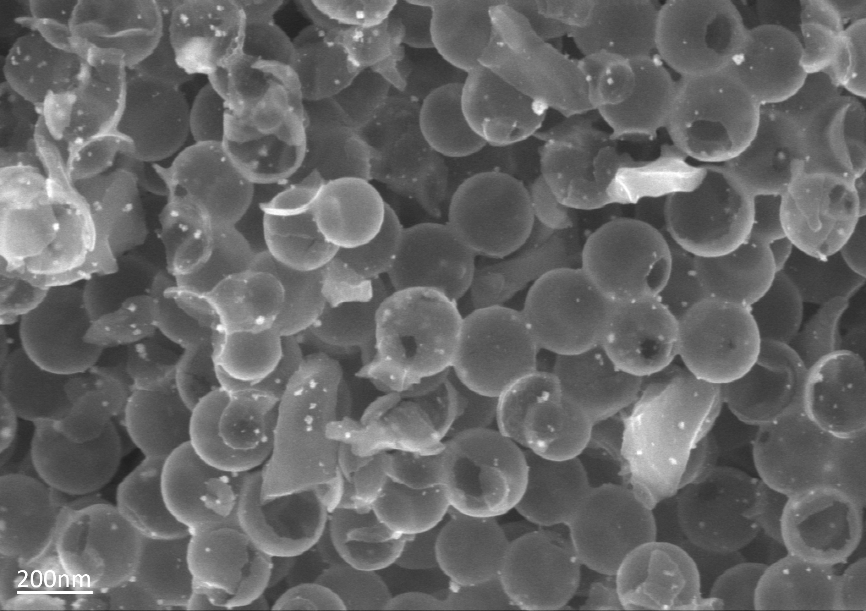


**Figure S6.** SEM image of Pt/B_10_@CB[7]/NHCSs.

**
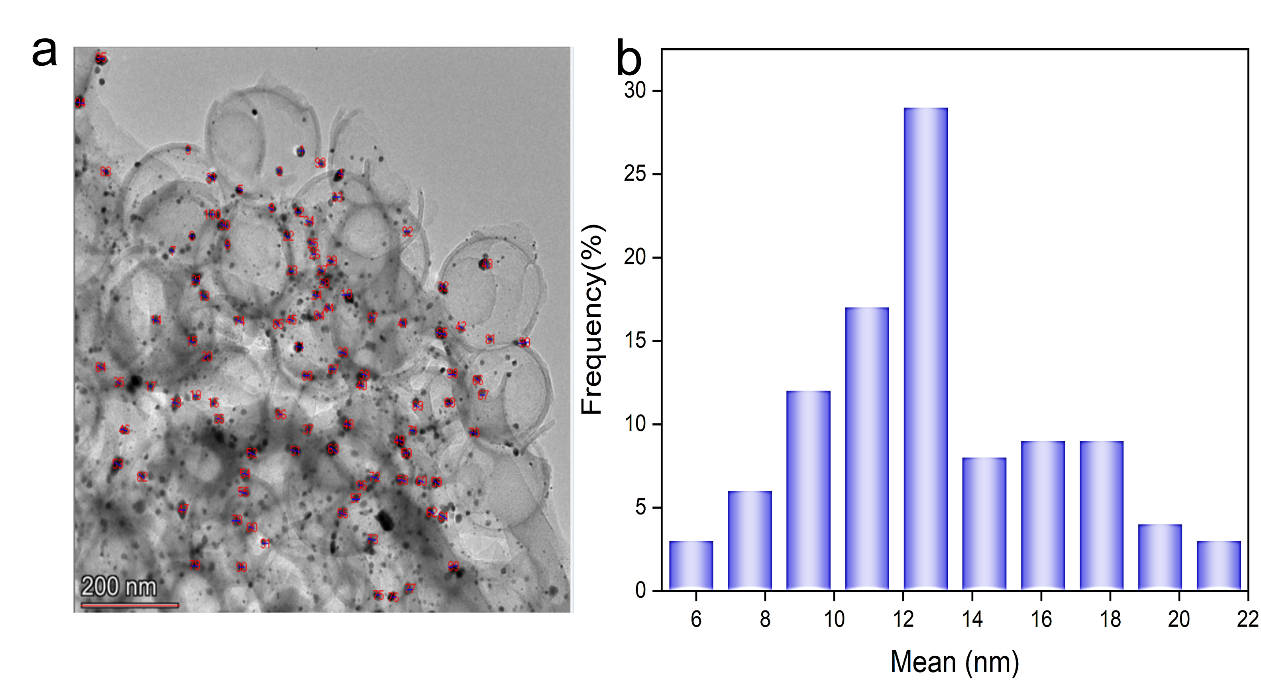
Figure S7.** (a) TEM images of Pt/BNHCSs; (b) Histogram of metal particle size statistics in (a).


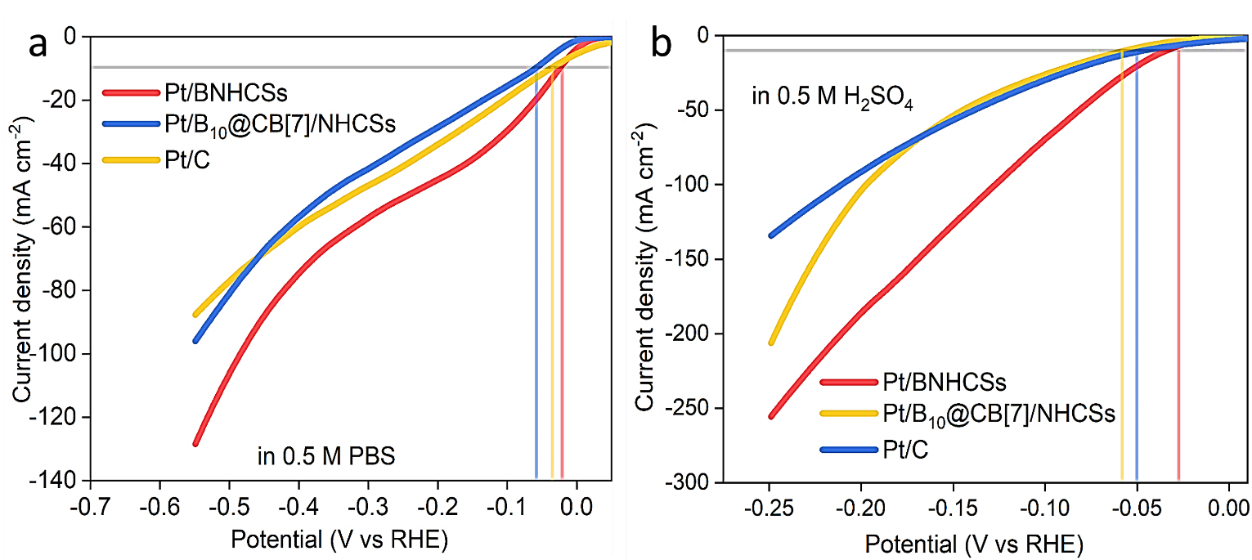
**Figure S8.** Using Pt/BNHCSs, Pt/B_10_@CB[7]/NHCSs, Pt/C as catalysts, the LSV curves of driving HER were collected in 0.5 M PBS (a) and 0.5 M H_2_SO_4_ (b). (Scan rate: 10 mV s^-1^)


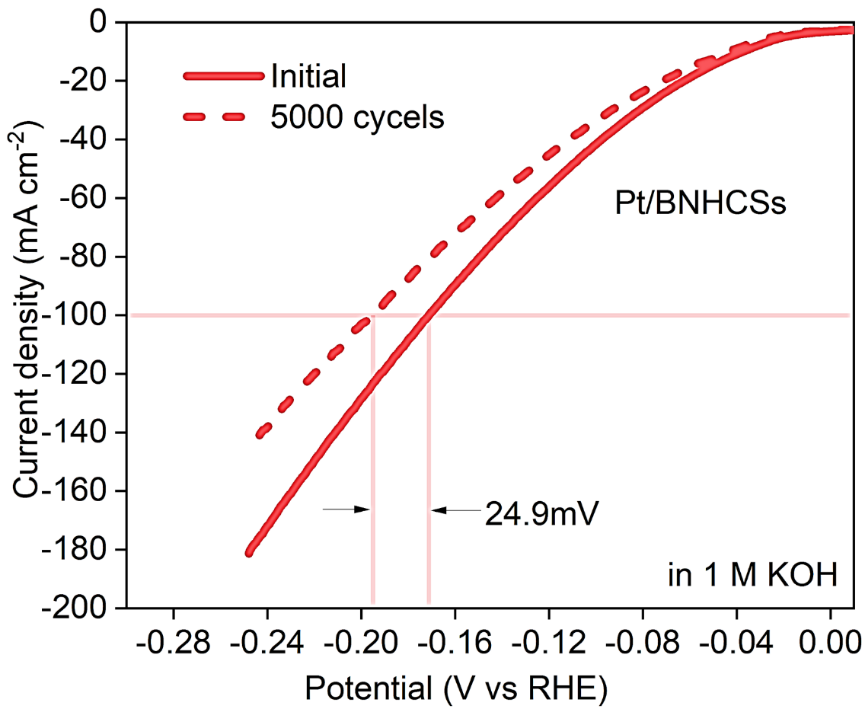


**Figure S9.** The stability of HER catalyzed by Pt/BNHCSs in 1 M KOH slution (by accelerated CV test).


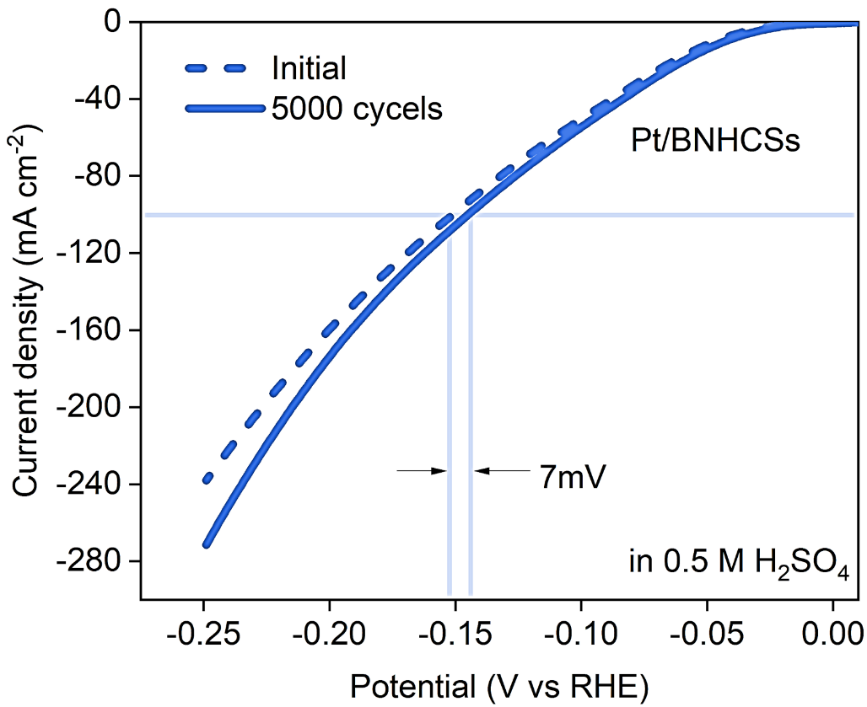


**Figure S10.** The stability of HER catalyzed by Pt/BNHCSs in 0.5 M H_2_SO_4_ (by accelerated CV test).


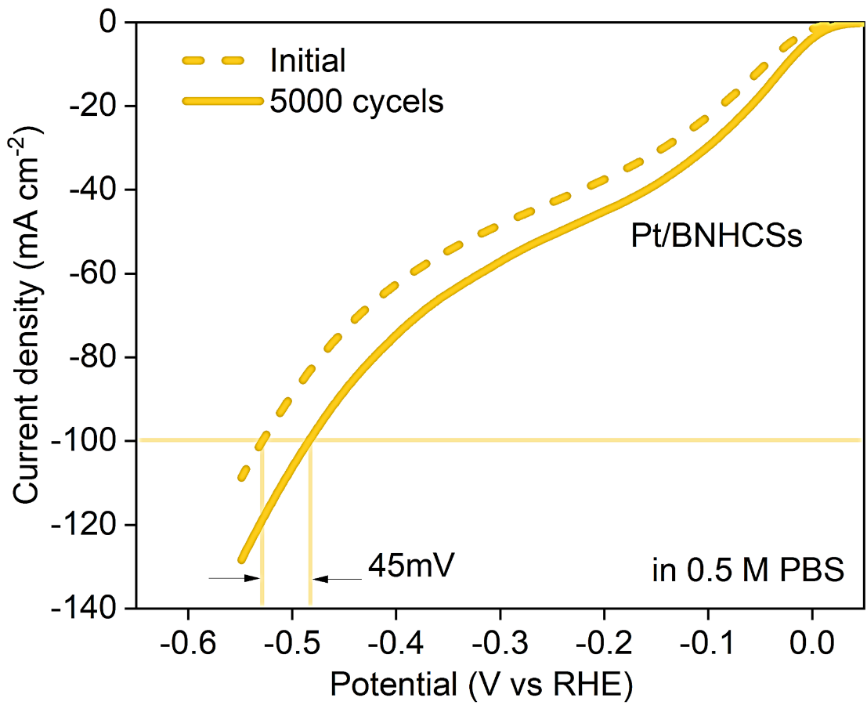


**Figure S11.** The stability of HER catalyzed by Pt/BNHCSs in 0.5 M PBS (by accelerated CV test).


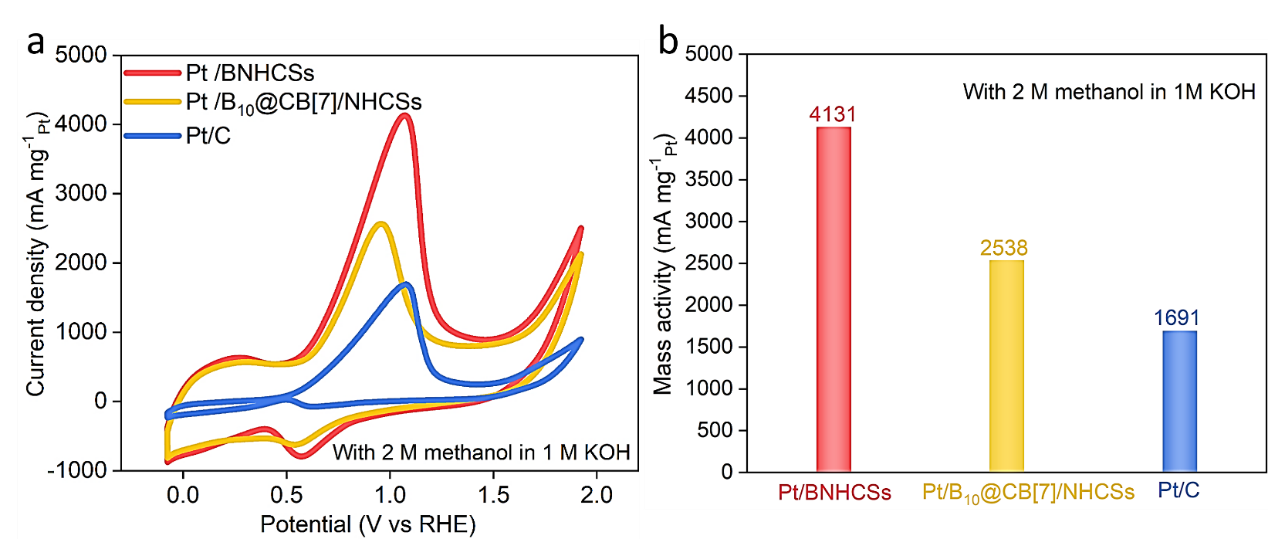
**Figure S12.** (a) The CV curves of MOR collection were driven by Pt/BNHCSs, Pt/B_10_@CB[7]/NHCSs and Pt/C as catalysts. (b) Mass activity of different catalysts for catalytic MOR.


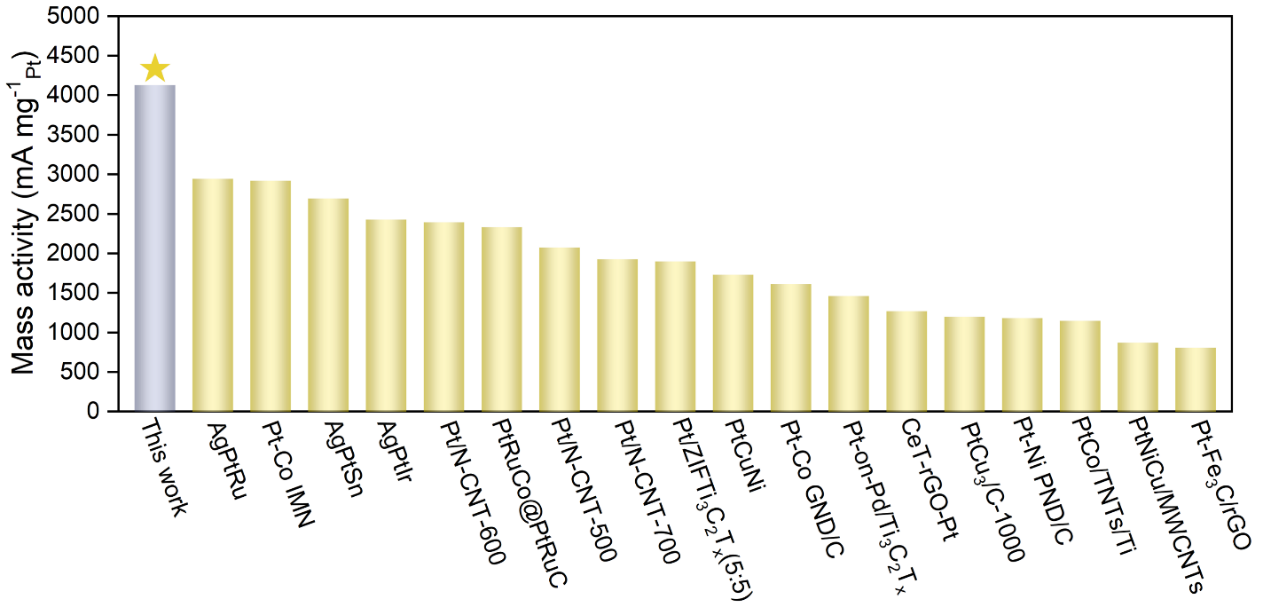


**Figure S13.** Comparison of Pt/BNHCSs catalytic MOR mass activity with other advanced Pt-based MOR catalysts previously reported in the literature.


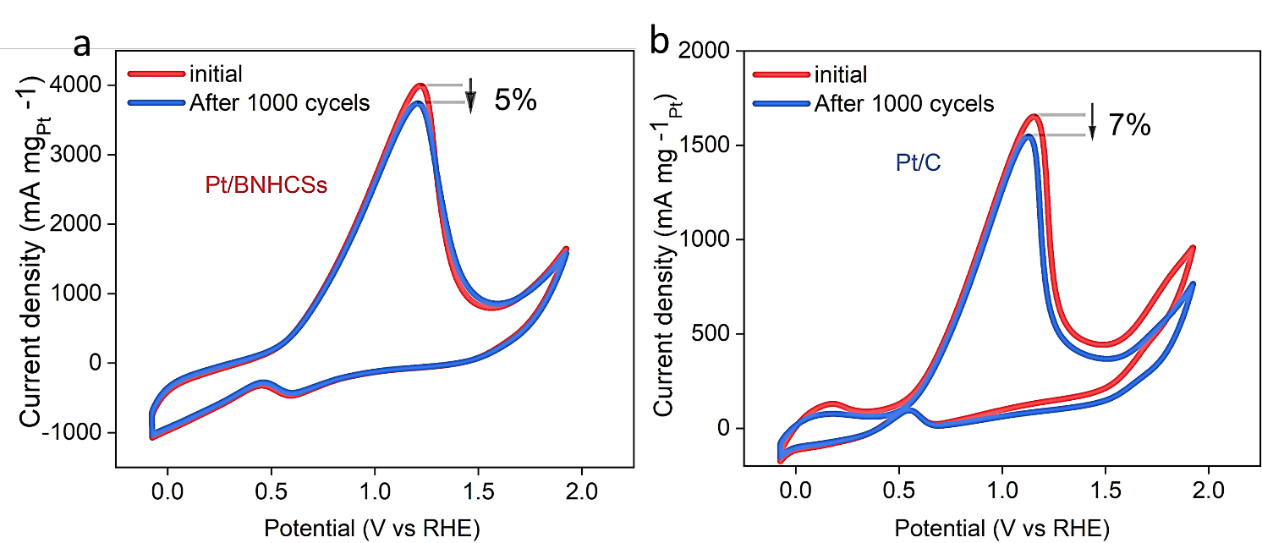


**Figure S14.** (a) The stability of MOR catalyzed by Pt/BNHCSs in alkaline medium (by accelerated CV test); (b) The stability of Pt/C catalyzed MOR in alkaline medium (by accelerated CV test).


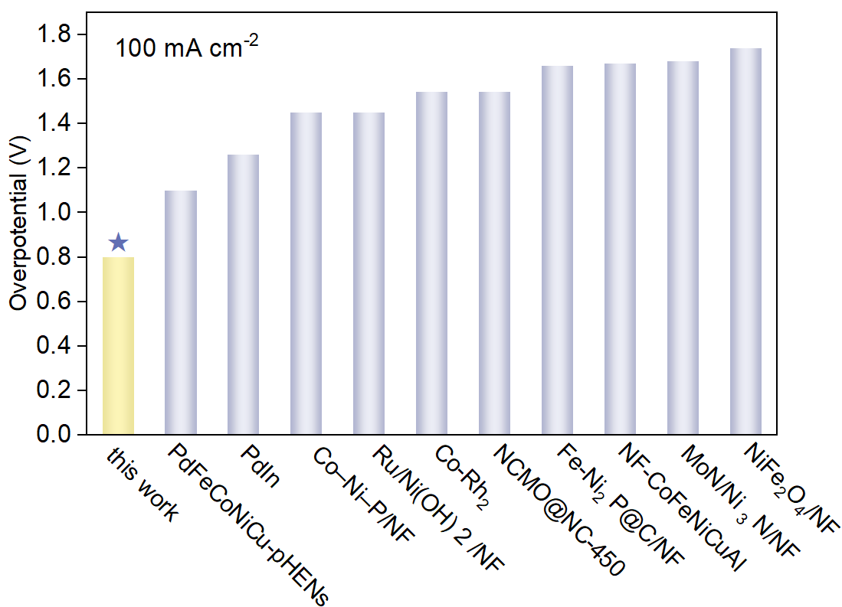


**Figure S15.** At a current density of 100 mA cm^-2^, the overpotential of Pt/BNHCSs as a catalyst for MOR-assisted electrolysis of water was compared with the catalyst reported in the previous literature.


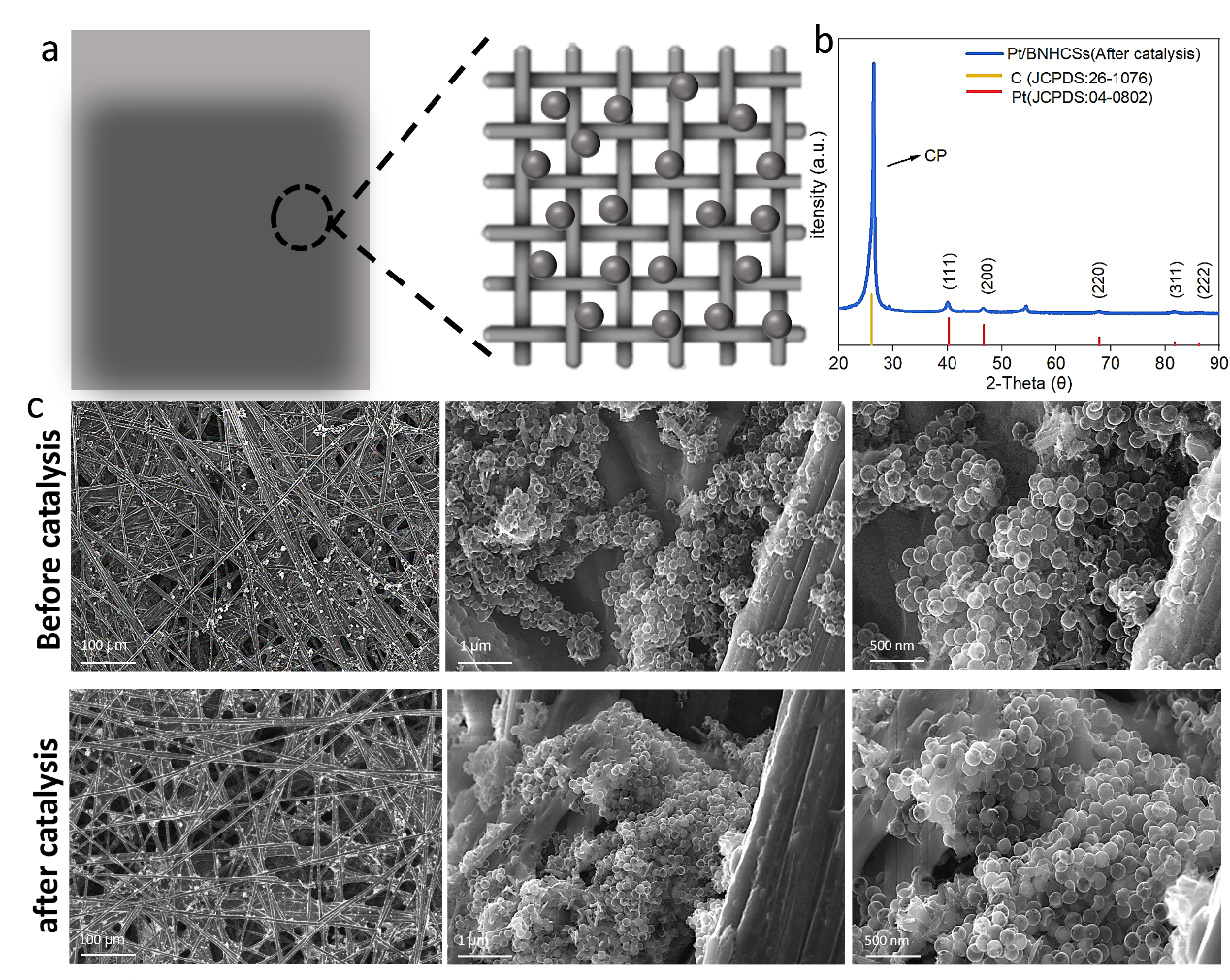


**Figure S16.** (a) The schematic diagram of Pt/BNHCSs ink coated on carbon paper; (b) The PXRD of Pt/BNHCSs long-term catalytic MOR; (c) SEM images of Pt/BNHCSs before and after long-term catalytic MOR.


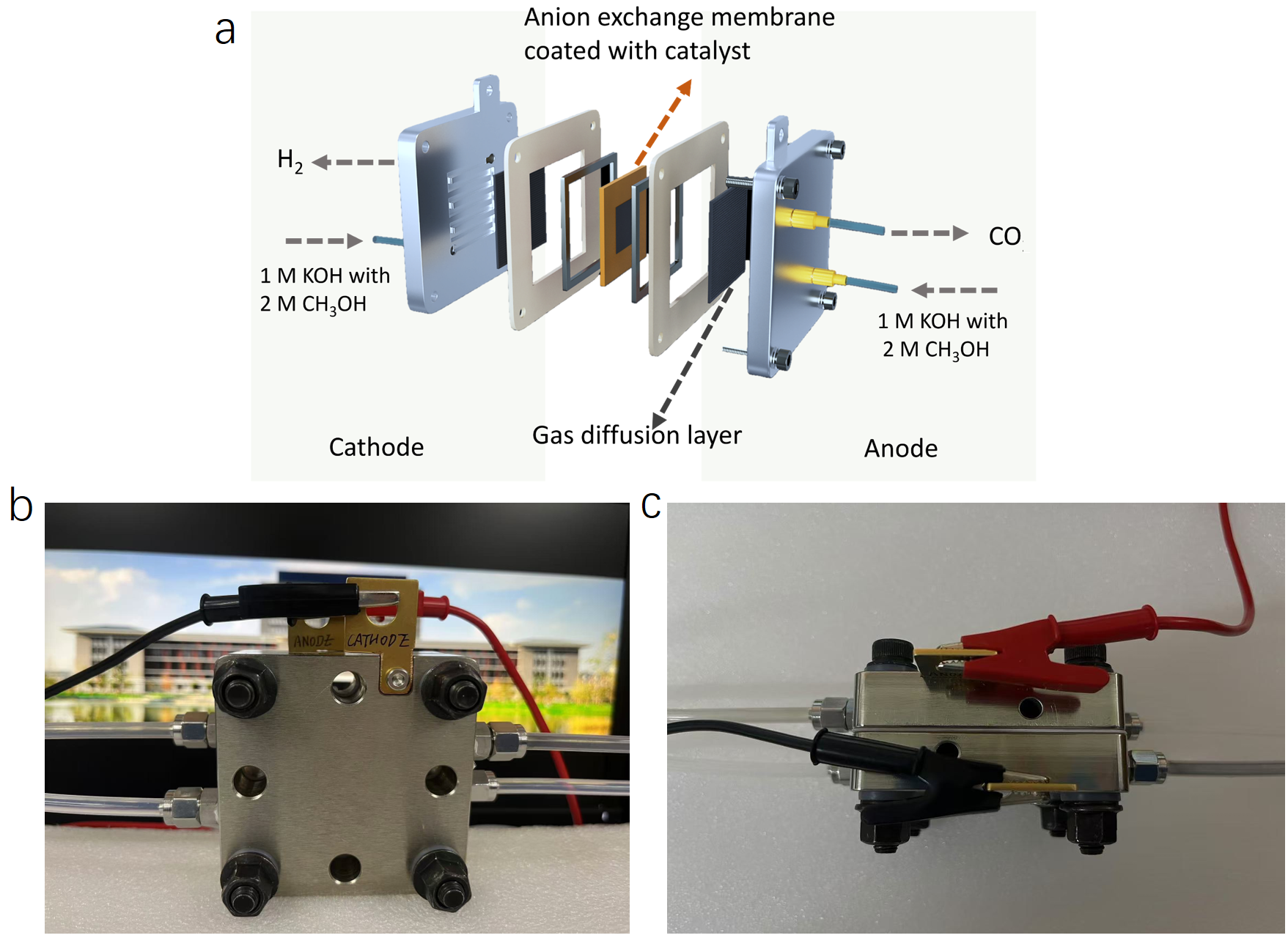


**Figure S17.** (a) Schematic diagram of MEA (membrane electrode assembly) catalytic HER and MOR; (b-c) Optical photo of HER and MOR catalyzed simultaneously by MEA.


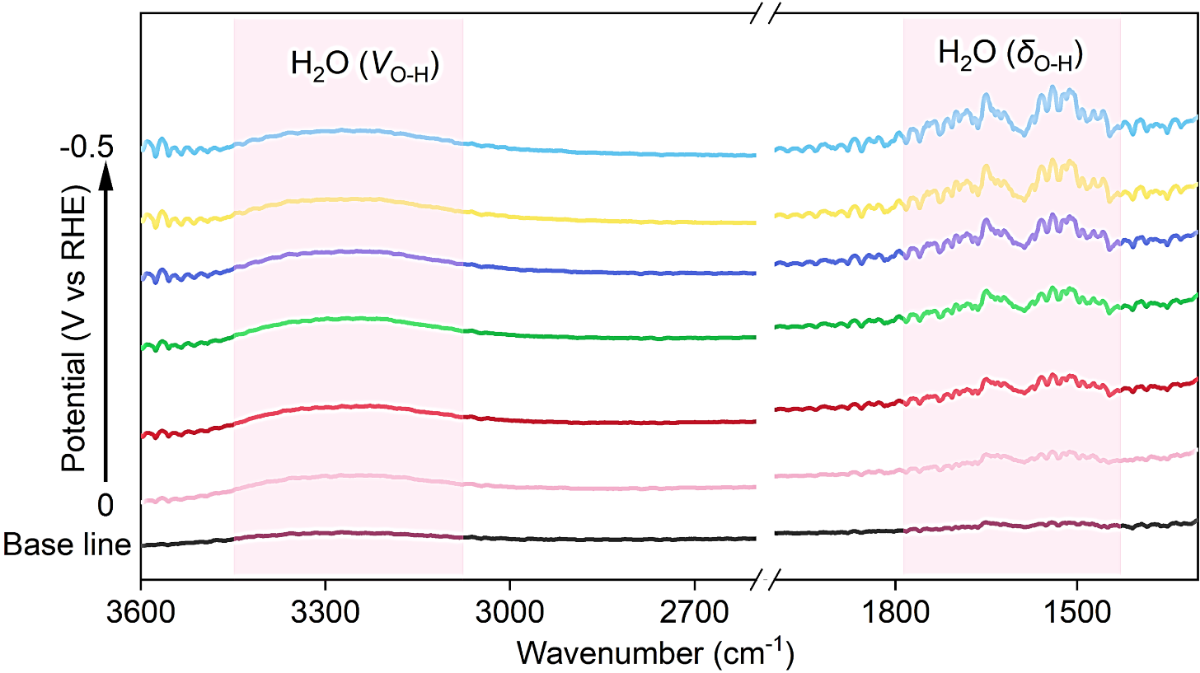


**Figure S18.** *In-situ* ATR-FTIR signals collected during the HER reaction process (Pt/BNHCSs as a catalyst).


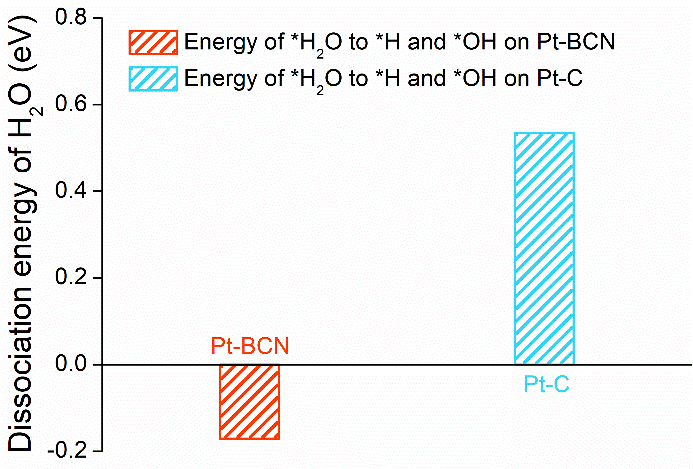


**Figure S19** Dissociation energies of H_2_O at Pt sites on Pt-BCN and Pt-C models.


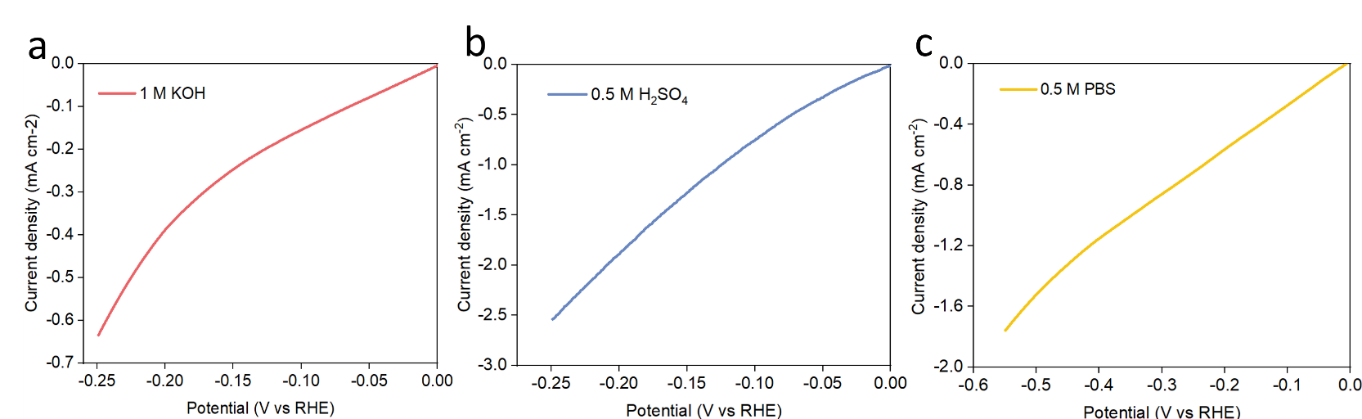
**Figure S20.** The LSV curves of HER driven by BNHCSs in alkaline electrolyte (a), acidic electrolyte (b) and neutral electrolyte (c) (scanning rate: 10 mV s^-1^).





**Figure S21.** The CV curves were collected in 1 M KOH containing 2 M methanol with Pt/BNHCSs and BNHCSs as catalysts (scan rate: 50 mV s^-1^).


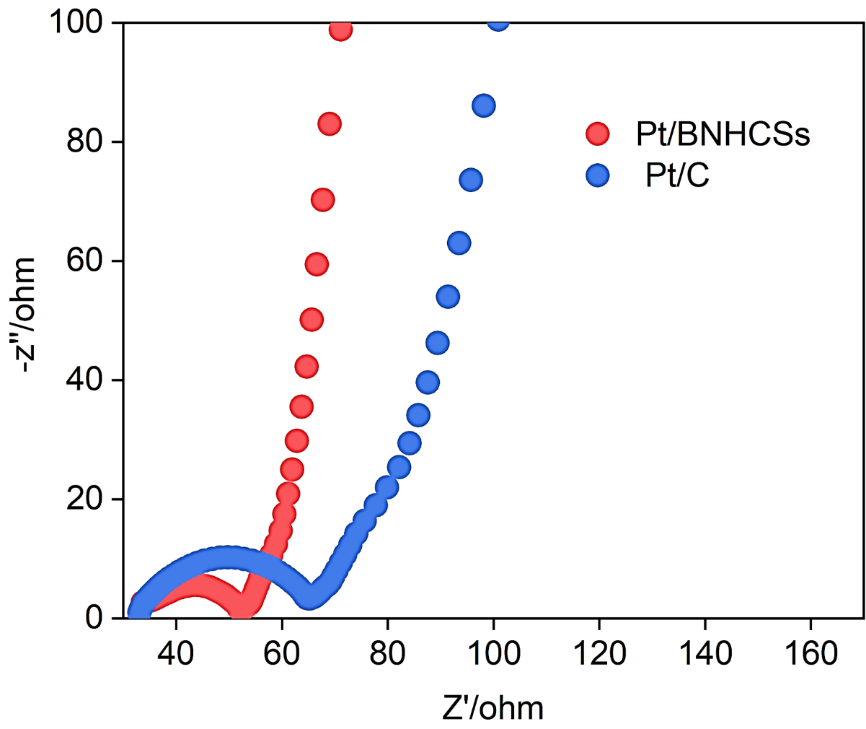


**Figure S22.** Nyquist plots of Pt/BNHCSs and commercial Pt/C (20 wt%).


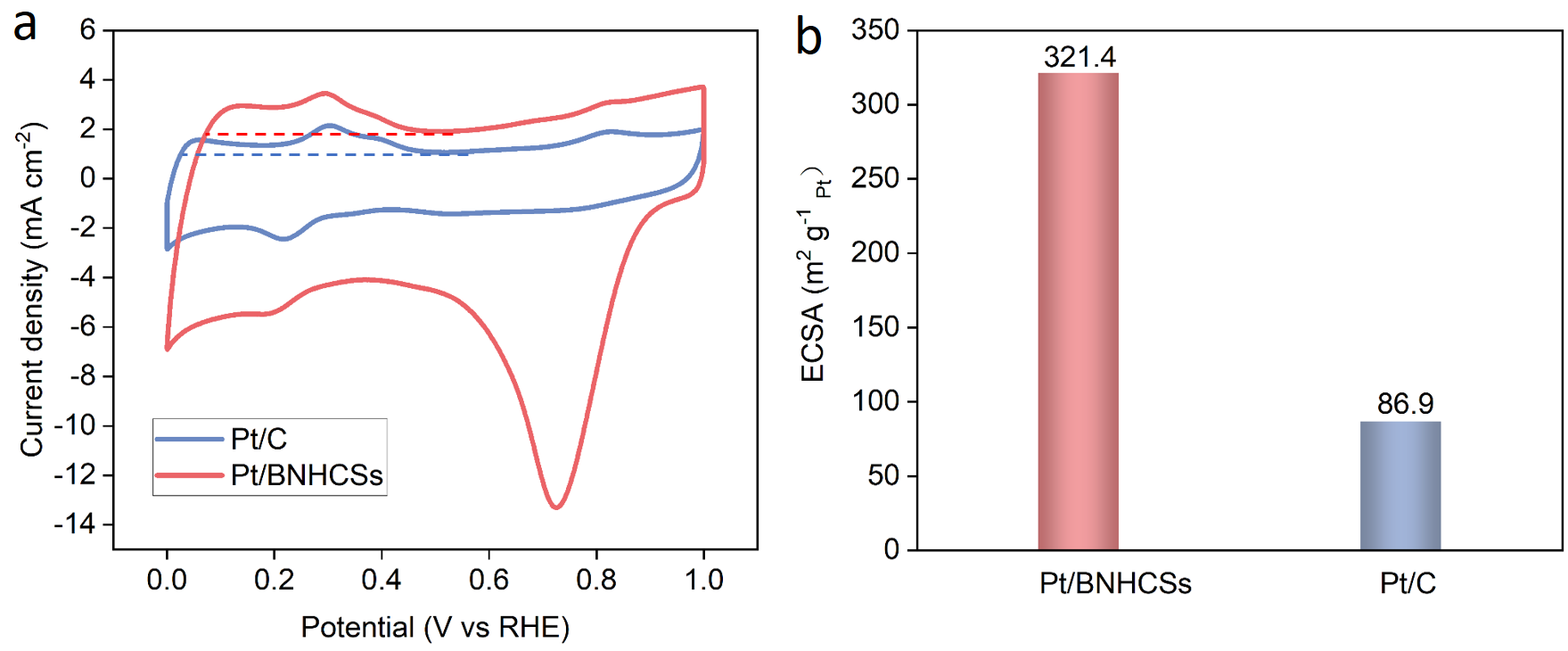


**Figure S23.** (a) The CV curves (scan rate 50 mV s^-1^) were collected in 1 M KOH solution when Pt/BNHCSs and Pt/C were used as working electrodes, respectively; (b) ECSA of Pt/ BNHCSs and Pt/C.


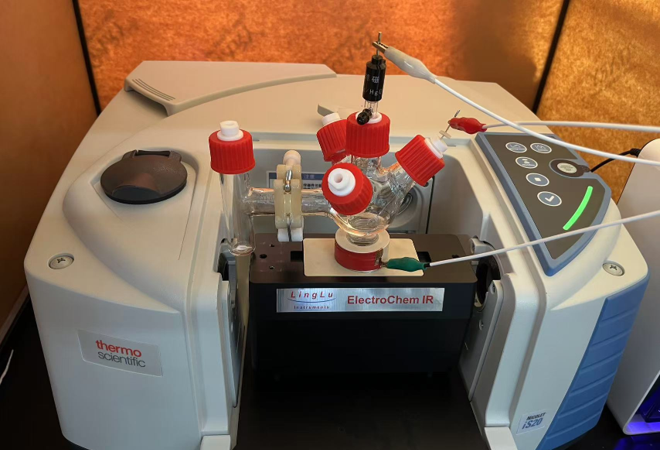


**Figure S24.** *In-situ* ATR-FTIR monitoring technology device.

**Table S1.** The overpotential and Tafel slope of Pt/BNHCSs, Pt/B_10_@CB[7]/NHCSs, Pt/C in different electrolytes were compared.

| **Catalyst** | **Electrolyte** | **Overpotential（mV）** | **Tafel slope**  **(mV dec^-1^)** |
| --- | --- | --- | --- |
| Pt/BNHCSs | 1 M KOH | 24.9 | 54.1 |
| Pt/B_10_CB[7]/NHCSs |  | 41.9 | 86.9 |
| 20 wt% Pt/C |  | 39.9 | 61.8 |
| Pt/BNHCSs | 0.5 M H_2_SO_4_ | 36 | 89 |
| Pt/B_10_CB[7]/NHCSs |  | 56 | 98 |
| 20 wt% Pt/C |  | 48 | 115.8 |
| Pt/BNHCSs | 0.5 M PBS | 24 | 38 |
| Pt/B_10_CB[7]/NHCSs |  | 59 | 49.23 |
| 20 wt% Pt/C |  | 38 | 95.32 |

**Table S2.** The catalytic performance of Pt/BNHCSs for HER was compared with previously reported catalysts in alkaline electrolyte at a current density of 10 mA cm^-2^.

| **Catalysts** | **Electrolyte** | **Overpotential**  **(mV)** | **Refs.** |
| --- | --- | --- | --- |
| Sr_2_RuO_4_ | 1 M KOH | 61 | ^[2]^ |
| Pt NPs@CF | 1 M KOH | 49 | ^[3]^ |
| Mo_2_C@NC@Pt | 1 M KOH | 47 | ^[4]^ |
| Rh_2_S_3_/NC | 1 M KOH | 38 | ^[5]^ |
| RuCo@NC-600 | 1 M KOH | 36 | ^[6]^ |
| Pt_5_Cu_2_ NTs | 1 M KOH | 34 | ^[7]^ |
| Rh_2_P | 1 M KOH | 30 | ^[8]^ |
| Pt/BNHCSs | 1 M KOH | 24.9 | This work |
| Ru@GnP | 1 M KOH | 22 | ^[9]^ |
| 2DPC-RuMo | 1 M KOH | 18 | ^[10]^ |
| PtRu/mCNTs | 1 M KOH | 15 | ^[11]^ |

**Table S3.** The HER catalytic performance of Pt/BNHCSs was compared with the previously reported catalysts at a current density of 10 mA cm^-2^ in an acidic electrolyte.

| **Catalysts** | **Electrolyte** | **Overpotential**  **(mV)** | **Refs.** |
| --- | --- | --- | --- |
| PATP/PtNPs | 0.5 M H_2_SO_4_ | 86.1 | ^[12]^ |
| Pt-MoC/NCT | 0.5 M H_2_SO_4_ | 74 | ^[13]^ |
| Ru/D-NPC | 0.5 M H_2_SO_4_ | 68 | ^[14]^ |
| Pt-VGNSAs/CC | 0.5 M H_2_SO_4_ | 60 | ^[15]^ |
| RuNi/CQDs | 0.5 M H_2_SO_4_ | 58 | ^[16]^ |
| Pt_3_Rh–Co_3_O_4_/C | 0.5 M H_2_SO_4_ | 55 | ^[17]^ |
| Pt-MoP-MoO/C | 0.5 M H_2_SO_4_ | 43 | ^[18]^ |
| Pt/CoFe_2_O_4_/NC | 0.5 M H_2_SO_4_ | 42 | ^[19]^ |
| Pt/RuCeOx-PA | 0.5 M H_2_SO_4_ | 41 | ^[20]^ |
| Rh_2_P/NPC | 0.5 M H_2_SO_4_ | 40 | ^[21]^ |
| Pt/BNHCSs | 0.5 M H_2_SO_4_ | 36 | This work |
| Mo_2_C@NC@Pt | 0.5 M H_2_SO_4_ | 27 | ^[4]^ |
| MOC-Ru | 0.5 M H_2_SO_4_ | 21 | ^[22]^ |
| Ru/β-Mo_2_C | 0.5 M H_2_SO_4_ | 18 | ^[23]^ |

| **Catalysts** | **Electrolyte** | **Overpotential**  **(mV)** | **Refs.** |
| --- | --- | --- | --- |
| Pt–TiO_2_−x NS | 1 M PBS | 88 | ^[24]^ |
| Pt-NiB@NF | 1 M PBS | 70 | ^[25]^ |
| Ru/D-NPC | 1 M PBS | 61 | ^[14]^ |
| RuCo@NC-600 | 1 M PBS | 60 | ^[6]^ |
| Pt/np-Co_0.85_Se | 1 M PBS | 55 | ^[26]^ |
| Pt NPs@CF | 1 M PBS | 53 | ^[3]^ |
| Pt_3_Fe/NMCS-A | 1 M PBS | 48 | ^[27]^ |
| Rh_2_S_3_/NC | 1 M PBS | 46 | ^[28]^ |
| BC_3_N@Pt | 1 M PBS | 38.5 | ^[29]^ |
| Pt-Pd@NPA | 1 M PBS | 34.8 | ^[30]^ |
| PtRu | 1 M PBS | 25 | ^[31]^ |
| Pt/BNHCSs | 0.5 M PBS | 24 | This work |
| RuNi/CQDs | 1 M PBS | 18 | ^[16]^ |
| PtRu/mCNT | 1 M PBS | 17 | ^[11]^ |

**Table S4.** In neutral electrolyte, the HER catalytic performance of Pt/BNHCSs was compared with that of previously reported catalysts at a current density of 10 mA cm^-2^.

**Table S5**. The mass activity of Pt/BNHCSs for MOR was compared with other advanced Pt-based catalysts reported in the literature.

| **Catalysts** | **Mass activity**  **(mA mg _Pt_^-1^)** | **Refs.** |
| --- | --- | --- |
| Pt/BNHCSs | 4131 | This work |
| AgPtRu | 2946 | ^[32]^ |
| Pt-CoIMN | 2920 | ^[33]^ |
| AgPtSn | 2694 | ^[32]^ |
| AgPtIr | 2428 | ^[32]^ |
| Pt/N-CNT-600 | 2394.7 | ^[34]^ |
| PtRuCo@PtRuC | 2333 | ^[35]^ |
| Pt/N-CNT-500 | 2073.7 | ^[34]^ |
| Pt/N-CNT-700 | 1926.3 | ^[34]^ |
| Pt/ZIFTi_3_C_2_Tx(5：5) | 1900.1 | ^[36]^ |
| PtCuNi | 1730 | ^[37]^ |
| Pt-CoGND/C | 1615 | ^[38]^ |
| Pt-on-Pd/Ti_3_C_2_Tx | 1461.7 | ^[39]^ |
| CeT-rGO-Pt | 1268.6 | ^[40]^ |
| PtCu_3_/C-1000 | 1200 | ^[41]^ |
| Pt-Ni PND/C | 1182 | ^[38]^ |
| PtCo/TNTs/Ti | 1148 | ^[42]^ |
| PtNiCu/MWCNTs | 871.4 | ^[43]^ |
| Pt-Fe_3_C/rGO | 808.5 | ^[44]^ |

**Table S6.** At a current density of 100 mA cm^-2^, the overpotential of Pt/BNHCSs catalyzed MOR-assisted electrolysis of water was compared with the catalysts reported in the previous literature.

| **Catalyst** | **Overpotential (V)**  **(100mA cm^-2^)** | **Refs.** |
| --- | --- | --- |
| Pt/BNHCSs | 0.81 | This work |
| PdFeCoNiCu-pHENs | 1.1 | ^[45]^ |
| PdIn | 1.26 | ^[46]^ |
| Co–Ni–P/NF | 1.45 | ^[47]^ |
| Ru/Ni(OH)_2_/NF | 1.45 | ^[48]^ |
| Co-Rh_2_ | 1.543 | ^[49]^ |
| NCMO@NC-450 | 1.544 | ^[50]^ |
| Fe-Ni_2_P@C/NF | 1.66 | ^[51]^ |
| NF-CoFeNiCuAl | 1.67 | ^[52]^ |
| MoN/Ni_3_N/NF | 1.68 | ^[53]^ |
| NiFe_2_O_4_/NF | 1.74 | ^[54]^ |

**Reference**

[1] W. Wang, X. Wang, J. Cao, J. Liu, B. Qi, X. Zhou, S. Zhang, D. Gabel, W. M. Nau, K. I. Assaf, H. Zhang, Chemical Communications 2018, 54, 2098.

[2] Y. Zhu, H. A. Tahini, Z. Hu, J. Dai, Y. Chen, H. Sun, W. Zhou, M. Liu, S. C. Smith, H. Wang, Z. Shao, Nature Communications 2019, 10, 149.

[3] Q. Li, Z. Deng, D. Tao, J. Pan, W. Xu, Z. Zhang, H. Zhong, Y. Gao, Q. Shang, Y. Ni, X. Li, Y. Chen, Q. Zhang, Advanced Functional Materials 2024, 34, 2411283.

[4] J.-Q. Chi, J.-Y. Xie, W.-W. Zhang, B. Dong, J.-F. Qin, X.-Y. Zhang, J.-H. Lin, Y.-M. Chai, C.-G. Liu, ACS Applied Materials & Interfaces 2019, 11, 4047.

[5] J. Li, G. Zhan, J. Yang, F. Quan, C. Mao, Y. Liu, B. Wang, F. Lei, L. Li, A. W. M. Chan, L. Xu, Y. Shi, Y. Du, W. Hao, P. K. Wong, J. Wang, S.-X. Dou, L. Zhang, J. C. Yu, J. Am. Chem. Soc. 2020, 142, 7036.

[6] F. Zhang, Y. Zhu, Y. Chen, Y. Lu, Q. Lin, L. Zhang, S. Tao, X. Zhang, H. Wang, Journal of Materials Chemistry A 2020, 8, 12810.

[7] H. Zhang, X. Guo, W. Liu, D. Wu, D. Cao, D. Cheng, Journal of Colloid and Interface Science 2023, 629, 53.

[8] F. Yang, Y. Zhao, Y. Du, Y. Chen, G. Cheng, S. Chen, W. Luo, Advanced Energy Materials 2018, 8, 1703489.

[9] F. Li, G.-F. Han, H.-J. Noh, I. Ahmad, I.-Y. Jeon, J.-B. Baek, Advanced Materials 2018, 30, 1803676.

[10] K. Tu, D. Tranca, F. Rodríguez-Hernández, K. Jiang, S. Huang, Q. Zheng, M.-X. Chen, C. Lu, Y. Su, Z. Chen, H. Mao, C. Yang, J. Jiang, H.-W. Liang, X. Zhuang, Advanced Materials 2020, 32, 2005433.

[11] B. Pang, X. Liu, T. Liu, T. Chen, X. Shen, W. Zhang, S. Wang, T. Liu, D. Liu, T. Ding, Z. Liao, Y. Li, C. Liang, T. Yao, Energy & Environmental Science 2022, 15, 102.

[12] J. Wang, J. Yu, J. Wang, K. Wang, L. Yu, C. Zhu, K. Gao, Z. Gong, Z. Li, R. Devasenathipathy, D. Cai, H. Xie, G. Lu, Small 2023, 19, 2207135.

[13] Z. Li, P. Yu, D. Shen, X. Zhang, Z. Liang, B. Wang, L. Wang, Chinese Chemical Letters 2024, 109713.

[14] W. Li, H. Zhang, K. Zhang, W. Hu, Z. Cheng, H. Chen, X. Feng, T. Peng, Z. Kou, Applied Catalysis B: Environmental 2022, 306, 121095.

[15] H. Zhang, W. Ren, C. Guan, C. Cheng, Journal of Materials Chemistry A 2017, 5, 22004.

[16] Y. Liu, X. Li, Q. Zhang, W. Li, Y. Xie, H. Liu, L. Shang, Z. Liu, Z. Chen, L. Gu, Z. Tang, T. Zhang, S. Lu, Angewandte Chemie International Edition 2020, 59, 1718.

[17] N. Bhuvanendran, C. W. Park, H. Su, S. Y. Lee, Environmental Research 2023, 229, 115950.

[18] J. Peng, Y. Shi, Z. Xiao, Y. Ye, Z. Li, S. Lin, Materials Research Bulletin 2023, 166, 112341.

[19] Q. Yan, B. Duolihong, B. Wang, X. Ma, X. Xia, International Journal of Hydrogen Energy 2024, 54, 1179.

[20] T. Liu, W. Gao, Q. Wang, M. Dou, Z. Zhang, F. Wang, Angewandte Chemie International Edition 2020, 59, 20423.

[21] S. Liu, Y. Chen, L. Yu, Y. Lin, Z. Liu, M. Wang, Y. Chen, C. Zhang, Y. Pan, Y. Liu, C. Liu, Journal of Materials Chemistry A 2020, 8, 25768.

[22] C. Yang, Z. Wu, Z. Zhao, Y. Gao, T. Ma, X. Luo, C. Cheng, Y. Wang, S. Li, C. Zhao, Advanced Materials 2023, 35, 2303331.

[23] Y. Li, Z. Dou, Y. Pan, H. Zhao, L. Yao, Q. Wang, C. Zhang, Z. Yue, Z. Zou, Q. Cheng, H. Yang, Nano Letters 2024, 24, 5705.

[24] K. M. Naik, E. Higuchi, H. Inoue, Nanoscale 2020, 12, 11055.

[25] X. Ma, Y. Zhang, L. Wu, Z. Huang, J. Yang, C. Chen, S. Deng, L. Wang, J. Chen, W. Hao, Journal of Materials Chemistry A 2024, 12, 33649.

[26] K. Jiang, B. Liu, M. Luo, S. Ning, M. Peng, Y. Zhao, Y.-R. Lu, T.-S. Chan, F. M. F. de Groot, Y. Tan, Nature Communications 2019, 10, 1743.

[27] P. Kuang, Z. Ni, B. Zhu, Y. Lin, J. Yu, Advanced Materials 2023, 35, 2303030.

[28] C. Zhang, H. Liu, Y. Liu, X. Liu, Y. Mi, R. Guo, J. Sun, H. Bao, J. He, Y. Qiu, J. Ren, X. Yang, J. Luo, G. Hu, Small Methods 2020, 4, 2000208.

[29] X. Zhao, M. Zheng, Z. Zhang, Y. Wang, Y. Zhou, X. Zhou, H. Zhang, Journal of Materials Chemistry A 2021, 9, 16427.

[30] C. Yang, H. Lei, W. Z. Zhou, J. R. Zeng, Q. B. Zhang, Y. X. Hua, C. Y. Xu, Journal of Materials Chemistry A 2018, 6, 14281.

[31] L. Li, G. Zhang, B. Wang, T. Yang, S. Yang, Journal of Materials Chemistry A 2020, 8, 2090.

[32] W.-C. Geng, A.-Q. Lou, S.-Y. Ma, K. Yao, Y.-Q. Xue, R.-Y. Yang, J.-J. Li, Journal of Alloys and Compounds 2024, 972, 172803.

[33] J. Chen, J. Dong, J. Huo, C. Li, L. Du, Z. Cui, S. Liao, Small 2023, 19, 2301337.

[34] Y. Li, H. Li, Y. Zhao, D. Ji, P. Guo, G. Li, X. Zhao, Small 2023, 19, 2303065.

[35] Z. Li, S. Ke, X. Zheng, Y. Huang, W. Fu, Y. Wang, Y. Nie, Chemical Engineering Journal 2024, 493, 152544.

[36] H. Huang, J. Qin, C. Liu, L. Luo, Y. Lan, L. Yang, J. Zhang, H. He, Carbon 2024, 226, 119171.

[37] Y. Ling, J. Wang, L. Zhuang, H. Yan, ACS Applied Energy Materials 2023, 6, 1965.

[38] X. Li, Y. Liu, J. Zhu, P. Tsiakaras, P. K. Shen, Journal of Colloid and Interface Science 2022, 607, 1411.

[39] C. Yang, Q. Jiang, H. Liu, L. Yang, H. He, H. Huang, W. Li, Journal of Materials Chemistry A 2021, 9, 15432.

[40] L. Kong, X. Chen, W. Zhu, Q. Ge, M. Bo, H. Wei, Journal of Alloys and Compounds 2023, 960, 170553.

[41] Z. Xing, J. Li, S. Wang, C. Su, H. Jin, Nano Research 2022, 15, 3866.

[42] Y. Chang, Y. Chen, Z. Yang, J. Wang, H. Li, Journal of Alloys and Compounds 2023, 968, 171859.

[43] Z. Wang, L. Wang, K. K. Rani, M. Waqas, D. Huang, Q. Huang, X. Liu, Z. Yang, X. Peng, D.-H. Chen, W. Chen, Y. Fan, Journal of Alloys and Compounds 2023, 946, 169354.

[44] G. Yang, X. Li, Y. Liu, J. Wang, S. Min, Y. Jiao, H. Yan, Journal of Materials Chemistry A 2024, 12, 16511.

[45] Y. Liu, W. Ding, J. Liu, G. Zhao, W. Li, Y. Liu, Journal of Materials Chemistry A 2024, 12, 30757.

[46] S. Yin, S. Liu, Z. Wang, Y. Xu, X. Li, H. Wang, L. Wang, Chemical Engineering Journal 2022, 435, 134711.

[47] X. Yue, S. Liping, H. Lihua, Z. Hui, ACS Applied Nano Materials 2023, 6, 10312.

[48] J. Lin, J. Chen, C. Tan, Y. Zhang, Y. Li, RSC Advances 2024, 14, 18695.

[49] Y. Guo, X. Yang, X. Liu, X. Tong, N. Yang, Advanced Functional Materials 2023, 33, 2209134.

[50] S. E. Islam, D.-R. Hang, C.-T. Liang, K. H. Sharma, H.-C. Huang, M. M. C. Chou, ACS Applied Energy Materials 2023, 6, 9543.

[51] D. Li, Z. Li, R. Zou, G. Shi, Y. Huang, W. Yang, W. Yang, C. Liu, X. Peng, Applied Catalysis B: Environment and Energy 2022, 307, 121170.

[52] J. Dang, H. Xu, X. Zhang, J. Jian, J. Huang, C. Zhang, Q. Wang, H. Miao, J. Yuan, Journal of Alloys and Compounds 2024, 1004, 175935.

[53] Z. Yang, K. Zhao, C. Shi, H. Ma, D. Yuan, Z. Yi, Journal of The Electrochemical Society 2024, 171.

[54] X. Du, M. Tan, T. Wei, H. Kobayashi, J. Song, Z. Peng, H. Zhu, Z. Jin, R. Li, W. Liu, Chemical Engineering Journal 2023, 452, 139404.
